# Learning the Structure of Biomedical Relationships from Unstructured Text

**DOI:** 10.1371/journal.pcbi.1004216

**Published:** 2015-07-28

**Authors:** Bethany Percha, Russ B. Altman

**Affiliations:** 1 Biomedical Informatics Training Program, Stanford University, Stanford, California, United States of America; 2 Departments of Medicine, Genetics and Bioengineering, Stanford University, Stanford, California, United States of America; University of Colorado School of Medicine, UNITED STATES

## Abstract

The published biomedical research literature encompasses most of our understanding of how drugs interact with gene products to produce physiological responses (phenotypes). Unfortunately, this information is distributed throughout the unstructured text of over 23 million articles. The creation of structured resources that catalog the relationships between drugs and genes would accelerate the translation of basic molecular knowledge into discoveries of genomic biomarkers for drug response and prediction of unexpected drug-drug interactions. Extracting these relationships from natural language sentences on such a large scale, however, requires text mining algorithms that can recognize when different-looking statements are expressing similar ideas. Here we describe a novel algorithm, Ensemble Biclustering for Classification (EBC), that learns the structure of biomedical relationships automatically from text, overcoming differences in word choice and sentence structure. We validate EBC's performance against manually-curated sets of (1) pharmacogenomic relationships from PharmGKB and (2) drug-target relationships from DrugBank, and use it to discover new drug-gene relationships for both knowledge bases. We then apply EBC to map the complete universe of drug-gene relationships based on their descriptions in Medline, revealing unexpected structure that challenges current notions about how these relationships are expressed in text. For instance, we learn that newer experimental findings are described in consistently different ways than established knowledge, and that seemingly pure classes of relationships can exhibit interesting chimeric structure. The EBC algorithm is flexible and adaptable to a wide range of problems in biomedical text mining.

## Author Summary

Virtually all important biomedical knowledge is described in the published research literature, but Medline currently contains over 23 million articles and is growing at the rate of several hundred thousand new articles each year. In this environment, we need computational algorithms that can efficiently extract, aggregate, annotate and store information from the raw text. Because authors describe their results using natural language, descriptions of similar phenomena vary considerably with respect to both word choice and sentence structure. Any algorithm capable of mining the biomedical literature on a large scale must be able to overcome these differences and recognize when two different-looking statements are saying the same thing. Here we describe a novel algorithm, Ensemble Biclustering for Classification (EBC), that learns the structure of drug-gene relationships automatically from the unstructured text of biomedical research abstracts. By applying EBC to the entirety of Medline, we learn from the structure of the text itself approximately 20 key ways that drugs and genes can interact, discover new facts for two biomedical knowledge bases, and reveal rich and unexpected structure in how scientists describe drug-gene relationships.

## Introduction

Biomedical research generates text at an incredible rate. Each year, several hundred thousand new articles enter Medline from over 5,500 unique journals [1, 2]. The literature’s rapid growth and the rise of interdisciplinary domains like bioinformatics and systems biology are changing how the scientific community interacts with this important resource. Knowledge bases like OMIM [3], DrugBank [4] and PharmGKB [5] manually curate and restructure information from the literature to increase its accessibility to researchers and clinicians. These knowledge bases capture cross-sectional “slices” of the literature, drawing connections among facts reported in different journals, at different times, and in different research domains. Often, they examine the literature in ways not easily captured by current indexing strategies, such as MeSH terms or key words.

As the literature grows and the information we need to extract increases in complexity, full manual curation of these knowledge bases is rapidly becoming infeasible. Progress in natural language processing (NLP) has encouraged the development of automated and semi-automated methods for enabling more efficient curation of biomedical text [6–9], especially as biomedical research begins to explore even larger text-based resources, such as electronic medical records (EMRs) [10, 11]. However, tasks that are simple for human readers, such as recognizing when two different-looking statements mean the same thing, or when one statement is a more general version of another statement, are often extremely challenging for NLP algorithms. One way around this problem is to infer the meaning of words and phrases by examining their usage patterns in large, unlabeled text corpora, an approach called “distributional semantics” [12–14]. If two words or phrases are used in similar contexts, they are likely to be semantically related.

Here we introduce a novel algorithm, called Ensemble Biclustering for Classification (EBC), that applies this strategy to uncover relationships between biomedical entities, such as drugs, genes and phenotypes. We focus on the problem of drug-gene relationship extraction and characterization from unstructured biomedical text, using statistical dependency parsing to extract descriptions of drug-gene relationships from Medline sentences and applying EBC to recognize when two drug-gene pairs share a similar relationship, even when they are described differently in the text. We show that EBC significantly improves our ability to extract both pharmacogenomic and drug-target relationships, and use it to discover new drug-gene relationships for PharmGKB and DrugBank. Finally, we combine EBC and hierarchical clustering to map the global “landscape” of drug-gene interactions, revealing much unforeseen complexity in how these relationships are described in text. We learn, for example, that there are subtle differences in how static knowledge (past discoveries) and new experimental discoveries are described, even when they refer to similar phenomena like inhibition, and that seemingly well-defined relationship classes (such as pharmacogenomic and drug-target relationships) often exhibit much more detailed chimeric structure than anticipated. More generally, we demonstrate that extracting biomedical relationships based on corpus-level usage patterns, rather than on the properties of individual sentences, helps bypass the need for large, annotated biomedical training corpora–an important property in a domain where few such corpora are available.

## Results

### Quantifying the variability of drug-gene descriptions in Medline sentences

The full set of abstracts from the 2013 edition of Medline contains approximately 184,000 sentences in which at least one drug name and at least one gene name are present. Many of these sentences contain multiple drug and gene names; the total number of unique drug-gene-sentence combinations is approximately 236,000.

**Fig 1:**
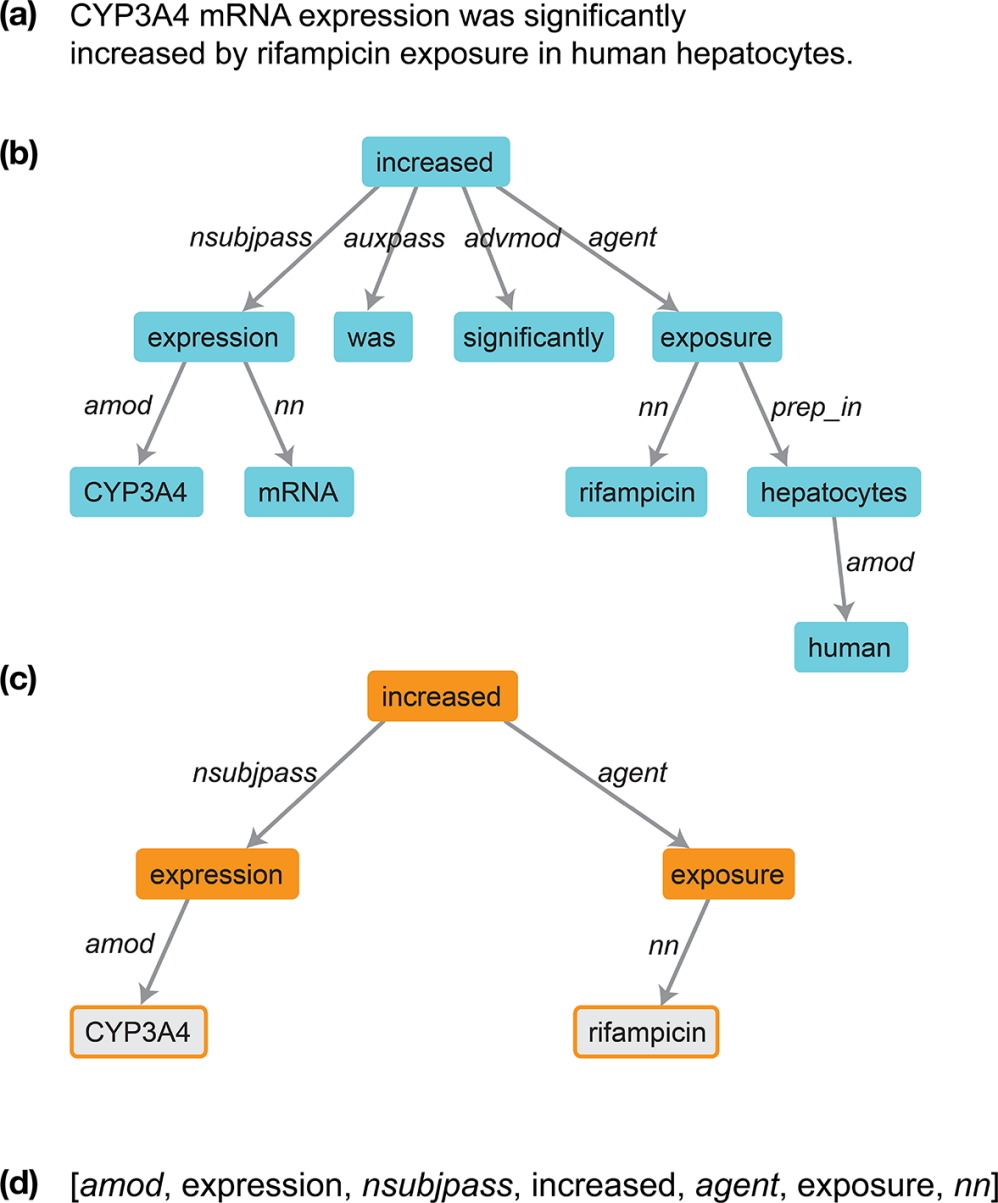
Example of a dependency graph for a Medline 2013 sentence. (a) The raw sentence. (b) The complete dependency graph for the sentence. (c) The dependency path connecting the gene CYP3A4 with the drug rifampicin. (d) A more compact representation of the dependency path.

As described in the Methods, we use dependency parsing to prune away irrelevant terms and phrases and focus attention on the parts of a drug-gene sentence most relevant to the relationship between a drug and a gene. The pruned versions of drug-gene sentences are called dependency paths. Fig 1 illustrates how dependency paths are constructed from raw sentences. Table 1 provides some common drug-gene dependency paths and associated example sentences. Details about the meanings of the individual grammatical dependencies, with examples, can be found in [15].

**Table 1:** Selected dependency paths and representative sentences.

	Dependency path	Example sentence (PubMed ID)	Frequency
[1]	[*appos*, inhibitor, *amod*]	**Geldanamycin** (GA), an **HSP90** inhibitor, is able to suppress 1,25-induced differentiation of HL60 cells. (20138989)	1181
[2]	[*appos*, inhibitor, *prep_of*]	The mNQO activity was insensitive to **dicoumarol**, a potent inhibitor of cytosolic **NQO1**. (10683249)	452
[3]	[*appos*, antagonist, *amod*]	The recommended therapy for stage III disease, based on clinical trials and by the Israeli Ministry of Health for 2006, includes **bosentan** (Tracleer), an **endothelin-1** antagonist. (18686806)	338
[4]	[*nsubjpass*, metabolized, *agent*]	**Amodiaquine** is mainly metabolized hepatically towards its major active metabolite desethylamodiaquine, by the polymorphic P450 isoform **CYP2C8**. (18855526)	204
[5]	[*nsubj*, inhibits, *dobj*]	**Salbutamol** inhibits **IFN-gamma** and enhances IL-13 production by PBMCs from asthmatics. (20523061)	118
[6]	[*nsubj*, inhibited, *dobj*, activity, *amod*]	**Clonidine** noncompetitively inhibited **acetylcholinesterase** activity in vitro and after in vivo administration at protective doses. (3761196)	73
[7]	[*appos*, antibody, *prep_against*]	**Trastuzumab**, a monoclonal antibody against **HER2**, has shown survival benefits when given with chemotherapy in all setting of HER2-positive breast cancer patients. (21129604)	71
[8]	[*nsubj*, increased, *dobj*, expression, *amod*]	**Carbachol** significantly increased **VEGF** expression in TMps, and this effect was totally reversed by methoctramine and pirenzepine. (15987429)	64
[9]	[*nsubj*, substrate, *prep_for*]	**Cyclosporin**, an immunosuppressant with a narrow therapeutic window, is a substrate for both **CYP3A4** and P-glycoprotein (Pgp). (12427482)	57
[10]	[*agent*, activated, *nsubjpass*]	These results suggest that **TRPV2** is specifically activated by **probenecid** and that this chemical might be useful for investigation of pain-related TRPV2 function. (17850966)	53
[11]	[*nsubj*, binds, *prep_to*]	**Pertuzumab** binds to **ErbB2** near the center of domain II, sterically blocking a binding pocket necessary for receptor dimerization and signaling. (15093539)	51
[12]	[*nsubj*, induces, *dobj*]	Tadalafil is mainly metabolized by cytochrome P450 (CYP) 3A4, and as **bosentan** induces **CYP2C9** and CYP3A4, a pharmacokinetic interaction is possible between these agents. (18305126)	30
[13]	[*nsubj*, increased, *dobj*, levels, *amod*]	When cells were cultured in a medium containing estrogen, **resveratrol** increased the **ErbB2** protein levels in a dose-dependent manner. (16488535)	29
[14]	[*prep_of*, metabolism, *prep_in*, involved, *nsubjpass*]	The results of preclinical studies demonstrated that **CYP3A4** is involved in the metabolism of **gefitinib** and that gefitinib is a weak inhibitor of CYP2D6 activity. (16176119)	21
[15]	[*nsubj*, inhibits, *dobj*, activation, *prep_of*]	**Imatinib** also inhibits the activation of **c-Abl**, which is a key downstream molecule of transforming growth factor-beta signaling, and PDGF receptors. (17603257)	17

The drug and gene names flanking each path are bolded. Some key abbreviations are listed here: *appos*: appositional modifier, *amod*: adjectival modifier, *prep*: prepositional modifier (if *prep_of*, the specific preposition used is “of”, if *prep_to*, it’s “to”, if *prep_for*, it’s “for”), *nsubjpass*: passive nominal subject, *agent*: complement of passive verb, *dobj*: direct object of active verb, *nsubj*: noun subject of active verb.

We can quantitatively estimate the diversity of drug-gene descriptions in Medline by considering the space of all unique drug-gene dependency paths. The vast majority of dependency paths are rare, indicating high variability in how drug-gene relationships are described. The total number of unique drug-gene dependency paths in Medline is approximately 197,000, of which 7,272 (4%) connect at least two different drug-gene pairs. The total number of unique drug-gene pairs co-occurring in Medline sentences is 49,564, of which 14,052 (28.4%) share a dependency path with at least one other drug-gene pair.

Table 2 describes the two datasets used in this paper, which consist of matrices, *M*, in which the rows are drug-gene pairs and the columns are dependency paths. A cell of *M*, *M*_*ij*_, contains “1” if drug-gene pair *i* is connected by dependency path *j* somewhere in Medline and “0” otherwise. Both of the datasets are over 99% sparse. An important goal, therefore, must be to recognize when different-looking statements are saying the same thing. Otherwise, we can only recognize that two drug-gene pairs share a relationship if their dependency paths are identical. The details of how EBC builds connections among different dependency paths can be found in the Methods.

**Table 2:** Summary of datasets for the PGx and drug-target relation extraction tasks. In the dense dataset, the drug-gene pairs and dependency paths represented must have occurred at least five times in Medline. In the sparse dataset, the dependency paths must have occurred at least twice, and all drug-gene pairs connected by these paths were included, even if they only occurred once.

Dataset	Task	Drug-gene pairs	Dependency paths	Nonzero matrix elements (sparsity)	Known relationships in dataset	Optimal row and column cluster numbers
Dense	PGx	3514	1232	10,007 (99.8%)	290	*k* = 30, *l* = 125
	Drug-target				410	
Sparse	PGx	14,052	7272	29,456 (99.97%)	545	*k =* 7, *l* = 25
	Drug-target				779	

### Identifying pharmacogenomic and drug-target relationships in biomedical text

We evaluated EBC’s ability to mine the literature for drug-gene pairs exemplifying two specific types of drug-gene relationships. The algorithm was given only the full, unlabeled text of Medline and a small number of drug-gene pairs that exemplified each type of relationship. We refer to the small sets of labeled drug-gene pairs (sizes 1, 2, 3, 4, 5, 10, 25, 50, and 100) as “seed sets”. No text was annotated and no specific sentences were marked as “evidence” for any particular type of relationship. The two relationship types we examined were:

1. **Pharmacogenomic (PGx) relationships.** PharmGKB’s relationships database [5] contains 6283 manually-curated drug-gene associations in which polymorphisms in the gene are known to impact drug response.
2. **Drug-target relationships.** DrugBank [4] maintains a list of known drug-gene relationships in which the protein product of the gene is a known target of the drug. This list contains 14,594 known relationships.

**Fig 2:**
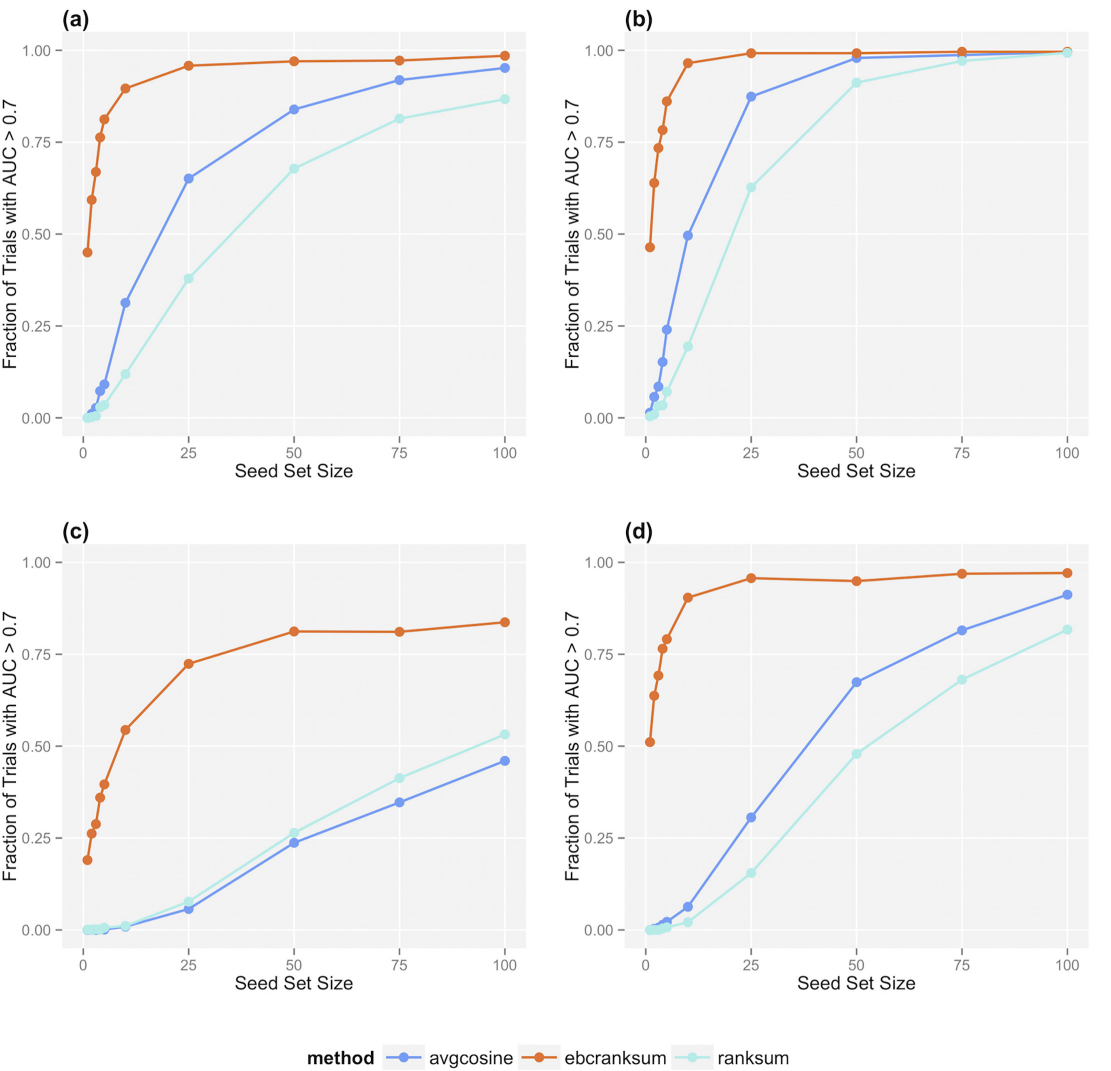
Classifier performance at the task of recognizing (a) PGx associations (dense matrix), (b) drug-target associations (dense matrix), (c) PGx associations (sparse matrix) and (d) drug-target associations (sparse matrix).

Fig 2 shows EBC’s performance extracting PGx and drug-target drug-gene pairs on the two datasets described in Table 2, and compares EBC to two alternative classifiers that do not account for the semantic relatedness of different dependency paths.

On both datasets, and on both tasks, EBC outperforms the other classifiers by a significant margin. On the dense dataset, using seed sets of only 10 labeled drug-gene pairs as input, EBC accurately (AUC > 0.7) ranks 89.6% of test sets for the PGx task and 96.5% of test sets for the drug-target task. In comparison, using the same seed and test sets, the best-performing non-EBC classifier accurately ranks only 31.3% of test sets for the PGx task and 49.6% for the drug-target task. On the sparse dataset, EBC’s increased performance is even more pronounced. Again using only 10 labeled pairs, EBC accurately ranks 54.4% of test sets on the PGx task and 90.4% on the drug-target task, compared to 1.1% and 6.3% for the best-performing non-EBC classifier.

EBC’s raw assessments of the similarity of all drug-gene pairs in both datasets can be found in S1 Data.

### Inferring connections among related descriptions based on patterns in the text

**Fig 3:**
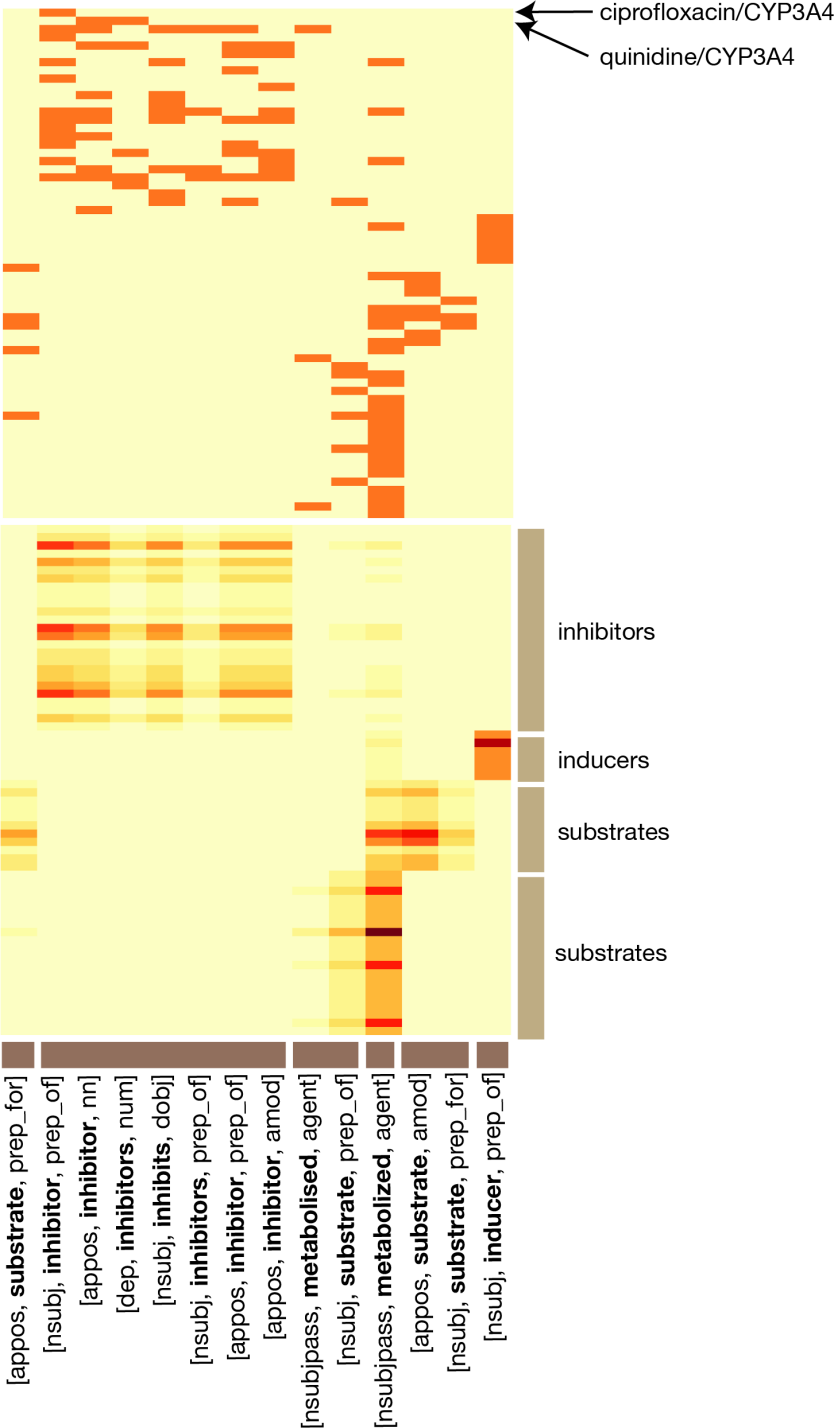
Example of ITCC output for a small matrix consisting of drug-CYP3A4 pairs and their associated dependency paths. The top heatmap shows the original data after the clustering was performed. An orange square represents an observed path (column) between a given drug-gene pair (row). The bottom heatmap shows the approximate distribution arising from a single ITCC run.

The backbone of EBC is a biclustering algorithm called Information-Theoretic Co-Clustering (ITCC; [16], see Methods). Fig 3 shows the result of one ITCC run on a small sample dataset consisting of dependency paths that connect different drugs to the gene CYP3A4 (a liver cytochrome involved in the pharmacokinetic pathways of many drugs) at least five times in Medline. This dataset contains 62 drug-gene pairs (where the gene is always CYP3A4) and 14 unique dependency paths. As with the datasets in Table 2, these are arranged in a matrix, *M*, where an element *M*_*ij*_ is “1” if drug-gene pair *i* is connected by path *j* somewhere in Medline, and “0” otherwise. We used ITCC to bicluster this matrix into four row clusters and six column clusters. Besides biclustering the matrix, ITCC produces a “smoothed” version of the matrix where certain elements that were not observed in the original dataset are filled in.

Fig 3 illustrates that the rows fragment into four clusters that reflect distinct ways that drugs can interact with CYP3A4. Row cluster 1 contains CYP3A4 inhibitors, a few of which are also substrates. Row cluster 2 contains CYP3A4 inducers. Row clusters 3 and 4 contain substrates of CYP3A4 that are not known inhibitors. EBC combines information from thousands of different biclusterings like this one to assess the relationship similarity of any two drug-gene pairs (rows) in the matrix, by looking at how frequently they cluster together.

It is also interesting to examine which columns of the matrix cluster together, as this provides insight into how the method is working. Fig 3 shows that the dependency paths naturally fragment into clusters reflecting known biomedical properties. All of the paths referring to inhibition, for example, appear together in column cluster 2. The sole path referring to induction appears by itself in column cluster 6. The other four clusters include paths describing situations where the drug is a substrate of CYP3A4, or is metabolized by it. We see a similar pattern emerge when we examine co-clustering frequencies of the columns on a larger dataset: the dense dataset from Table 2. Table 3 shows some dependency paths from this dataset that frequently cluster together over 2000 separate runs of ITCC. Paths that frequently cluster together appear to be semantically related.

**Table 3:** Some dependency paths that cluster together with relatively high frequency.

First Pattern	Second Pattern	Frequency of co-clustering
[*nsubj*, antibody, *partmod*, directed, *prep_against*]	[*nsubj*, antibody, *partmod*, targeting, *dobj*]	0.59
*D* is an antibody directed against *G*.	*D* is an antibody targeting *G*.	
[*prep_such_as*, inhibitor, *amod*]	[*prep_including*, inhibitors, *amod*]	0.31
*G* inhibitor such as *D*	*G* inhibitors, including *D*	
[*prep_such_as*, agonists, *nn*]	[*amod*, activators, *nn*]	0.12
*G* agonists, such as *D*,.* *.* *.	*G* activators, *D* and. . .	
[*nsubjpass*, metabolized, *agent*]	[*dep*, substrates, *nn*]	0.11
*D* is metabolized by *G*	*G* substrates (*D*,. . .),. . .	
[*nsubj*, blocked, *dobj*, activation, *amod*]	[*nsubj*, inhibited, *dobj*]	0.07
*D* blocked *G* activation	*D* inhibited *G*	
[*nsubj*, increased, *dobj*, expression, *prep_of*, mRNA, *nn*]	[*nsubj*, induces, *dobj*, activity, *amod*]	0.03
*D* increased the expression of *G* mRNA	*D* induces *G* activity	

The first line of each row shows the dependency path, the second an example of what that path would look like in the raw text. The symbol *D* represents the drug and *G* represents the gene.

### Mapping the semantic landscape of drug-gene interactions

**Fig 4:**
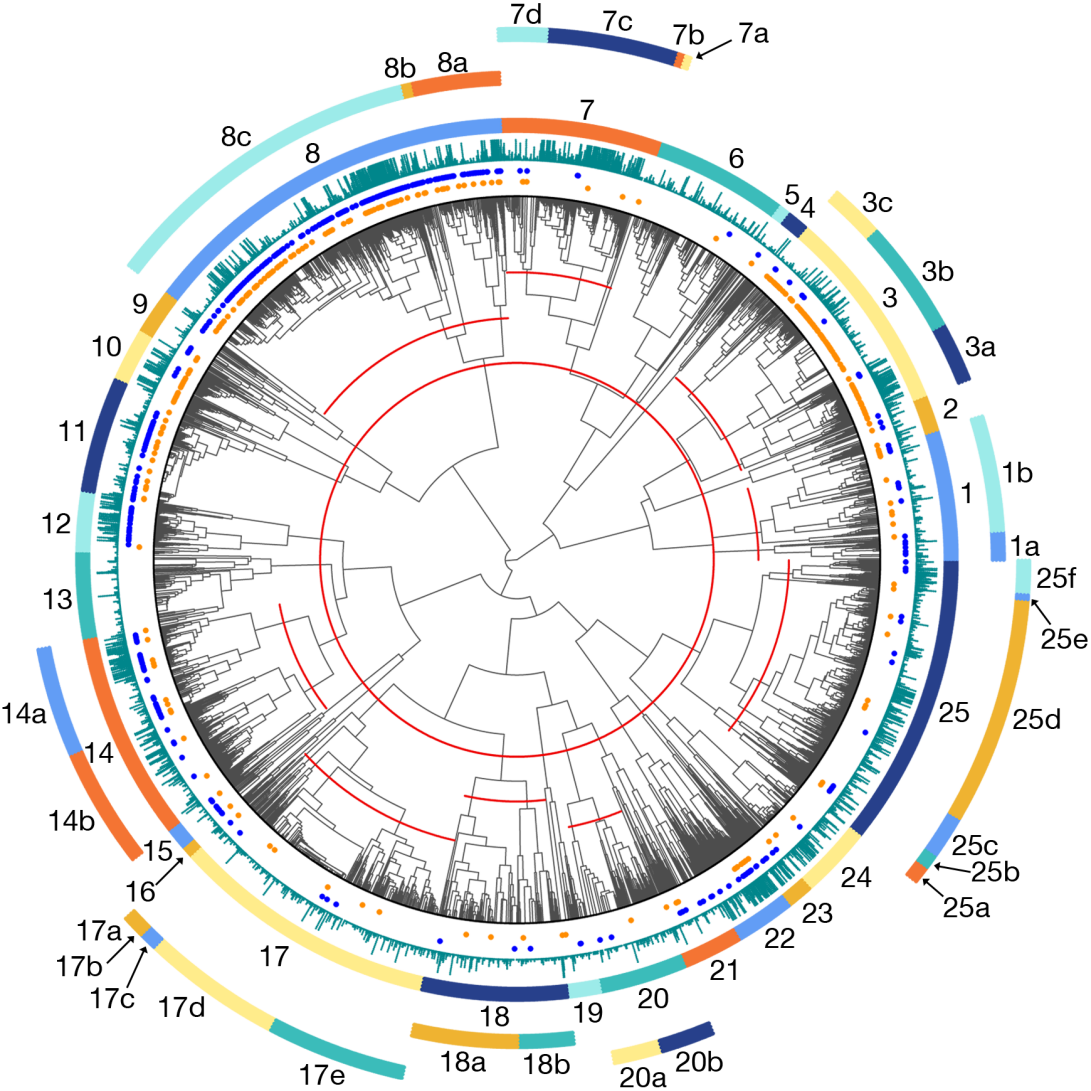
Dendrogram illustrating the semantic relationships among 3514 drug-gene pairs. In this dendrogram, the leaves represent 3514 drug-gene pairs that co-occur in Medline sentences at least 5 times, and we have cut the dendrogram at various levels (illustrated by the red lines in the interior of the dendrogram) to produce the colored clusters shown around the edges. Drug-gene pairs that are known drug-target relationships from DrugBank are denoted by blue dots, and those that are known PGx relationships from PharmGKB are denoted by orange dots. The heights of the turquoise bars are proportional to how often the corresponding drug-gene pairs co-occur in Medline sentences (a proxy for how well-documented they are).

EBC provides a measure of relationship similarity between every drug-gene pair and every other pair (the frequency with which each pair of rows in the data matrix cluster together). By combining these assessments with hierarchical clustering, we created the dendrogram shown in Fig 4, the details of which are described in the figure caption. Table 4 summarizes the general “themes” of the clusters from Fig 4 and includes the size of each cluster and the density of known PGx and drug-target relationships within that cluster. The cluster assignments for different slices of the dendrogram are provided in S3 Data.

**Table 4:** Explanation of the clusters shown in Fig 4. Clusters with 20 or fewer members are not described in the table in the interest of space.

	Theme	Cluster size	Key word/phrase	Example drug-gene pair	% PGx	% Drug-Target	Comment
**1a**	Synthesis	34	synthase	aldosterone, P450aldo	0.0	17.6	Many of the drugs in this cluster are endogenous compounds.
	*11 beta-Hydroxylase (P45011 beta) and aldosterone synthase (P450aldo) were situated in the inner mitochondrial membrane of the zona fasciculata-reticularis cells and in that of the zona glomerulosa cells*, *respectively*. *(9617077)*						
**1b**	Activation	134	increased activity	curcumin, caspase-8	9.0	6.7	In this cluster, activation is frequently associated with phosphorylation.
	*Curcumin also stimulated the activity of caspase-8*, *which initiates Fas signalling pathway of apoptosis*. *(11396178)*						
**2**	Enzyme activity	45	activity	estradiol, E2DH	6.7	6.7	The gene is typically an enzyme that chemically modifies the drug. A few transporter pairs are also present, such as (ornithine, ORNT1).
	*A fraction of the estradiol 17 beta-oxidoreductase (E2DH) activity in the vesicle remained associated to the membrane after disruption and treatment with 2 M NaCl*. *(3459941)*						
**3a**	Substrates	64	substrate	aminopterin, hOAT1	29.7	7.8	Relatively few mentions of “metabolism” compared to 3b and 3c. Reference to transporters such as P-gp, hOAT1, SERT.
	*These findings show that both aminopterin and methotrexate are substrates of hOAT1 and hOAT3*, *and that there are differences between the antifolates in terms of their transport characteristics*. *(20460822)*						
**3b**	Metabolism	131	metabolized	rosiglitazone, CYP2C8	37.4	0.8	Frequent reference to liver cytochromes such as CYP3A4 and CYP2D6.
	*Rosiglitazone*, *a thiazolidinedione antidiabetic medication used in the treatment of Type 2 diabetes mellitus*, *is predominantly metabolized by the cytochrome P450 (CYP) enzyme CYP2C8*. (15606443)						
**3c**	Substrates that (often) also affect activity	70	substrate	efavirenz, CYP2B6	37.1	5.7	The drug-gene pairs in this sub-cluster are mentioned together less frequently in the literature than those in 3a or 3b.
	*Efavirenz is extensively metabolized by CYP2B6*, *and associations between CYP2B6 polymorphisms and plasma efavirenz exposure have been reported*. (20639527) *Our results confirm that efavirenz induces CYP2B6 enzyme activity in vivo*, *as demonstrated by an increase in bupropion hydroxylation after 2 weeks of efavirenz administration*. (18989234)						
**4**	Third party involvement	28	Inhibits… to/by	rapamycin, PHAS-I	3.6	3.6	All of the drug-gene pairs in this cluster are connected by exactly one path, and the paths are unusual. They often refer to the involvement of a third molecule of some kind, raising the possibility of three-way interactions among drugs and genes.
	*Rapamycin may inhibit translation initiation by increasing PHAS-I binding to eIF-4E*. (7629182)						
**6**	Coadministration	172	in presence of	sunitinib, IFN-alpha	0.6	0.6	This cluster illustrates the blurry line between drugs and genes (proteins) since many drugs (in this case, IFN-alpha) are also proteins.
	*Herein*, *we report the results of a phase I dose-finding study of sunitinib in combination with IFN-alpha as first-line treatment in patients with metastatic RCC*. (19213665)						
**7c**	Increased production	141	induced, production, increase	PGE2, VEGF	1.4	1.4	Cluster 7 is distinguished by the presence of many proteins that act as drugs. These include IL-2, gp120, and PGE2.
	*These findings raise the possibility that endogenous PGE2 stimulates VEGF and bFGF mRNA expression in Mueller cells in vivo under conditions in which PGE2 production is increased*, *such as in injury*. (9501870)						
**7d**	Raised levels	52	levels, production	cisplatin, Rad51	5.8	3.8	Similar in theme to 7a-c, descriptions from this cluster involve drugs that raise protein levels. Sentences mostly report experimental results.
	*In addition*, *gefitinib decreased cisplatin- or MMC-elicited Rad51 protein levels by increasing Rad51 protein instability*. (18544565)						
**8a**	Antagonists	101	antagonist, blocker	plerixafor, CXCR4	11.9	39.6	Cluster 8 references inhibition more generally. EBC learns that antagonism (cluster 8a) is a subclass of inhibition.
	*Plerixafor is a selective antagonist of CXCR4 used for mobilization of hematopoietic stem cells (HSCs) for autologous stem cell transplantation (SCT) in patients with multiple myeloma (MM) and non-Hodgkin lymphoma (NHL)*. (19748593)						
**8c**	Inhibition	380	inhibitor of, inhibits	sildenafil, PDE5	18.7	37.9	Cluster 8c is large and includes some interesting smaller subclusters, such as antibodies against particular proteins, and inhibition, specifically, of protein activity or phosphorylation.
	*Although active sites of PDEs are apparently structurally similar*, *PDE4 is specifically inhibited by selective inhibitors such as rolipram*, *while PDE5 is preferentially blocked by sildenafil*. (15224132)						
**9**	Specific drug-protein interactions	56	target, kinase, protein	hyaluronate, GHAP	3.6	14.3	These are pairs where the protein is named for its function, which involves a particular action on the drug in question. In the second sentence, the pair is pyridoxal/Pdxk.
	*Cells were probed with the glial hyaluronate binding protein (GHAP) which was itself then visualized by conventional indirect immunofluorescence*. (2070821) *Transcriptome profiling revealed pyridoxal kinase (Pdxk) as a target gene of PAR bZip proteins in both liver and brain*. (15175240)						
**10**	Inhibitors *and* substrates	70	inhibitor, substrate, metabolized	verapamil, P-gp	30.0	4.3	Many drugs act as both inhibitors and substrates of proteins, including ritonavir/CYP3A4, quinidine/P-gp, and omeprazole/CYP2C19, all found in cluster 10.
	*It has been reported that verapamil and atorvastatin are inhibitors of both P-glycoprotein (P-gp) and microsomal cytochrome P450 (CYP) 3A4*, *and verapamil is a substrate of both P-gp and CYP3A4*. (18193210)						
**11**	Inhibition	148	inhibitor of; *G* inhibitors, such as *D*; inactivator	miglitol, alpha-glucosidase	12.2	27.0	There is little difference in meaning between this cluster and cluster 8c, except that there are variations in phrasing that are more common to one or the other cluster.
	*alpha-Glucosidase inhibitors*, *such as miglitol*, *are drugs that have greater affinity towards this enzyme in comparison to carbohydrates*. (19563873)						
**12**	Receptors	80	receptor(s), gene, antagonist	urokinase, uPAR	1.3	32.5	Cluster 12 contains a subcluster primarily composed of antagonist pairs, and a larger subcluster involving pairs where the gene is described as the “receptor” for the drug.
	*The urokinase receptor urokinase-type plasminogen activator receptor (uPAR) is a surface receptor capable of not only focalizing urokinase-type plasminogen activator (uPA)—mediated fibrinolysis to the pericellular micro-environment but also promoting cell migration and chemotaxis*. (22285761)						
**13**	Activation	112	activated, increased expression	simvastatin, Rac1	0.0	0.0	This is the largest cluster with zero representation of either PGx or drug-target relationships. The pair in the second sentence is estradiol/HO-1.
	*The small GTPase Rac1 was activated by simvastatin*, *and this was required for both PKB activation and IL-1beta secretion*. (18684863) *Estradiol increased HO-1 expression by 2- to 3-fold*, *an effect blocked by SU5416*, *and PPT mimicked the effects of estradiol on HO-1*. (20644008)						
**14a**	Agonists	129	agonist, hormone, analog	sumatriptan, 5-HT1B	7.0	33.3	
	*We compared the vasoconstrictor effects of 5-HT with those of the selective 5-HT1B/1D-receptor agonists sumatriptan and rizatriptan in human isolated cranial (middle meningeal) arteries*. (9862247)						
**14b**	Activation / stimulation	138	activates, induced, stimulates	resveratrol, AMPK	1.4	4.3	Focus is similar to cluster 13 but notably, there is relatively little reference to expression.
	*Moreover*, *resveratrol activated AMPK and inhibited phosphorylation of 4E-BP1 and S6 in diabetic rat kidneys*. (20332614)						
**15**	Protein binding	28	binds to; binding to	glibenclamide, SUR1	7.1	35.7	
	*ATP*, *in the presence of an ATP-regenerating system to oppose hydrolysis during incubation*, *inhibited glibenclamide binding to SUR1 and SUR2B (Y1206S) by approximately 60%*, *to SUR2A (Y1206S) by 21%*. (12145099)						
**17d**	Experimental methods	151	treatment, concentration, toxicities, mice, cells	dasatinib, STAT3	1.8	2.4	This cluster includes many sentences describing observed effects on expression/activity, but not as many as other nearby clusters. Cluster 17d is also home to one insidious error: the term “DLTS'' (“dose-limiting toxicities'') identified as a gene.
	*We hypothesized that the reactivation of STAT3 after dasatinib treatment represents the engagement of a compensatory signal for cell survival that blocks the antitumor effects of SFK inhibition*. (17634553) *Treatment of cultured cells from WT or Delta 18 COX-2 mice with flurbiprofen*, *which blocks substrate-dependent degradation*, *attenuates COX-2 degradation*, *and treatment of normal mice with ibuprofen increases the levels of COX-2 in brain tissue*. (19758985)						
**17e**	Effect on expression	148	investigate effect on *G* expression; alter, affect, decrease, regulated	colchicine, MEFV	1.3	0.0	If directionality of effect is reported in cluster1 17e, it is most often inhibition.
	*To investigate the effect of colchicine (the main therapeutic agent for FMF patients) and certain inflammatory cytokines (IL-1 beta*, *TNF-alpha*, *IFN-alpha*, *IFN-gamma) on MEFV expression and C5a inhibitor activity in neutrophils and primary peritoneal fibroblast cultures*. (11802319)						
**18a**	Induction of expression	123	increased/induced expression	imatinib, CXCR4	1.6	1.6	Typically experimental results reporting a positive effect of the drug on gene expression.
	*In KBM5 and K562 cells*, *imatinib*, *INNO-406*, *or IFN-alpha increased CXCR4 expression and migration*. (18202009)						
**18b**	Effect on expression, usually induction	65	by expression, inducer of, was induced by	melatonin, bcl-2	1.5	1.5	In many sentences, we know only that the effect of the drug on the expression of the gene was investigated. If directionality of effect is reported, it is most often induction.
	*Melatonin given before the ischemia enhanced the expression of bcl-2 in the penumbra area and had no significant effect on the expression of bax*. (10678086)						
**19**	Inhibition of activation	41	inhibited / suppressed activation (of *G*)	fluvastatin, NF-kappaB	4.9	4.9	This is another set of three-way interactions where the drug is suppressing activation of the protein by some other molecule.
	*Interestingly*, *fluvastatin suppressed IFN-gamma-induced NF-kappaB activation in parallel with p38 MAPK phosphorylation*. (19594754)						
**20a**	Effect on expression, usually inhibition	54	expression by, expression of, inhibited expression, decreased, reduced	montelukast, iNOS	0.0	3.7	There is a fairly even split in this cluster between methods and results.
	*This study investigated the effects of montelukast (a leukotriene receptor antagonist) on iNOS expression and activity in a Brown Norway (BN) rat allergic inflammation model and in L2 lung epithelial cell*. (14559427)						
**20b**	Decreased levels	59	decreased levels, inhibited expression, suppression	gefitinib, Rad51	1.7	0.0	Note that the example sentence here is identical to that in cluster 7d, but the drug in question is different. This single sentence describes two separate relationships with different characters.
	*In addition*, *gefitinib decreased cisplatin- or MMC-elicited Rad51 protein levels by increasing Rad51 protein instability*. (18544565)						
**21**	Inhibited activity / expression	76	inhibited activity, inhibited expression	minocycline, MMP-2	3.9	10.5	Focus is experimental observations, as opposed to stated prior knowledge (the dominant theme in cluster 8c).
	*Intraperitoneal minocycline at 45 mg/kg concentration twice a day (first dose immediately after the onset of reperfusion) significantly reduced gelatinolytic activity of ischemia-elevated MMP-2 and MMP-9 (p < 0*.*0003)*. (16846501)						
**22**	Inhibition	78	inhibited; *G* inhibitors, such as. . .	trastuzumab, HER2	10.3	17.9	There are some subtle differences between cluster 22 and cluster 8. Most notably, cluster 22 never references antagonism. Cluster 22 also contains some descriptions that never occur in cluster 8, such as “inhibited induction of” and “inhibited activation”. Similarly, cluster 8 contains some descriptions (besides those of antagonists) that never occur in cluster 22, such as “inhibitors of *G*, such as. . .”, “decreased activity”, and “inhibit activity”.
	*The humanized anti-HER2 monoclonal antibody trastuzumab inhibits the activation of HER2 and its multiple downstream signaling pathways*, *including the Ras/mitogen-activated protein kinase pathway*. (18451248)						
**23**	Protein binding (and) affects activity	33	activity, protein, binds	gp120, DC-SIGN	0.0	12.1	This small cluster actually contains two smaller subclusters, one of which focuses on protein activity and the other on binding. The descriptions of these drug-gene pairs include some different variants of those in clusters 15 and 25f.
	*gp120 additionally binds to DC-SIGN*, *a C-type lectin expressed on immature dendritic cells*. (11825572) *Moreover*, *exposure of hippocampal neurons to dexamethasone significantly increased caspase-3 activity*, *which was inhibited by co-treatment with agmatine*. (16777341)						
**24**	Patients with disease (error)	92	treatment, patients, disease	glyburide, NIDDM	3.3	2.2	This cluster illustrates one problem associated with using simple string matching to lexicons to identify drugs and genes: COPD and NIDDM are both gene names. Notably, however, these types of errors are “quarantined” together in the dendrogram.
	*140 NIDDM patients being treated with either glyburide (n = 70) or glipizide (n = 70) were randomly selected from the populations of patients receiving either drug using computerized pharmacy records*. (1421641)						
**25c**	Affects secretion / release	50	secretion	octreotide, calcitonin	0.0	0.0	Genes (proteins) in this cluster are generally hormones or cytokines, such as gastrin, lactogen, IL-1RA, and IL-13.
	*The inhibitory effect of octreotide on rGRF-induced calcitonin secretion was partially abolished by pretreating the cells with pertussis toxin*. (1355052)						
**25d**	Expression	252	on expression, by expression, inhibited / increased expression	indomethacin, MCP-1	2.0	2.0	The directionality of the drug's effect on expression varied within this cluster. The sentences mostly report experimental findings.
	*We found that*, *in murine podocytes*, *expression of monocyte chemoattractant protein 1 (MCP-1) in response to TNF-alpha was suppressed by indomethacin but not by ibuprofen*. (18799549)						
**25f**	Affects activity	38	activity, on activity	amitriptyline, EAAT3	2.6	10.5	
	*Our results suggested that amitriptyline at clinically relevant concentrations reversibly reduced EAAT3 activity via decreasing its maximal velocity of glutamate transporting function*. (19405995)						

Cluster 8, the largest cluster, contains drug-gene pairs whose descriptions mainly refer to inhibition. This cluster is highly enriched for both PGx and drug-target relationships. When cluster 8 is subdivided by cutting the dendrogram at a lower height, a subcluster (8a) of antagonists and their protein targets splits off from the main cluster. EBC has learned that antagonism is a subclass of inhibition. Cluster 10, which is a close relative of cluster 8 in the dendrogram, contains drug-gene pairs where the drug is both an inhibitor and a substrate of the protein, such as verapamil/P-glycoprotein.

Cluster 3, another large cluster, is almost exclusively devoted to metabolism and substrate relationships, and is highly enriched for PGx relationships, though not drug-target relationships. Cluster 3 contains three subclusters with slightly different properties. Cluster 3a involves mainly substrate relationships where the concept of “metabolism'' is not mentioned. These include, for example, transport relationships like aminopterin/hOAT1. Cluster 3b contains most of the metabolic relationships, many of which involve liver cytochromes like CYP3A4 and CYP2D6. Cluster 3c includes substrate relationships where the drug is often also described as having an effect on the activity of the protein.

Other clusters enriched for drug-target relationships include cluster 12, where the protein is described as the receptor for the drug, cluster 14a, where the drug is described as an agonist of the protein, and cluster 15, which refers to protein binding. Notably, cluster 14a (agonists) is part of a larger cluster, cluster 14, that encompasses activation and stimulation relationships. Here, EBC has learned that agonism is a subclass of activation. Interestingly, cluster 14b, the part of cluster 14 that refers to activation more broadly and does not specifically refer to agonism, is not enriched for drug-target relationships.

Clusters 1–16, which comprise 3 of the 4 main high-level groups within the dendrogram, are relatively easy to interpret: in general, each displayed a consistent theme. Clusters 17–25, however, involve descriptions of experimental methods or results about drug effects on gene expression or protein activity. Here, the dendrogram reveals a distinction between past and present knowledge. Drug-gene pairs that are already well-studied are often reported in a static context–“D is an inhibitor of G”, or “D is a G agonist”–whereas other pairs are reported primarily in an experimental context–“we investigated the effect of D on G expression”, “G was activated by D”, or “exposure to D significantly increased G activity”. Depending on the relative frequency of different types of descriptions, a drug-gene pair exemplifying an inhibitory relationship might end up in cluster 8 (mostly static descriptions) or cluster 21 (mostly experimental descriptions). Interestingly, drug-gene pairs from cluster 21 appear together in the literature significantly fewer times than drug-gene pairs from cluster 8 (median 9 times for cluster 21 vs. 16 times for cluster 8; maximum 66 times for cluster 21 vs. 2722 times for cluster 8; p < 0.0001, Mann-Whitney test), which seems to corroborate our assertion that the drug-gene pairs from cluster 21 represent more tentative experimental findings as opposed to well-established static knowledge.

Finally, the dendrogram reveals that PGx and drug-target relationships do not constitute distinct classes of relationships, but are chimeras. PGx relationships are composed of relatively distinct subgroups corresponding to (a) situations where the drug inhibits the gene/protein (and therefore, mutations in the gene could be expected to impact response to the drug), and (b) situations where the protein is involved in the metabolism or transport of the drug. Drug-target relationships overlap with (a) but not (b), and include other non-PGx subclasses, such as receptor binding and agonism.

### Discovering novel relationships for PharmGKB and DrugBank

EBC reliably detects new drug-gene pairs reflecting relationships of interest to PharmGKB and DrugBank, so we attempted to discover new examples from our corpus. We built seed sets containing all known relationships from PharmGKB and DrugBank and incorporated these into EBC to rank the remaining drug-gene pairs according to EBC’s certainty that they represented PGx or drug-target relationships. There was 13.6% overlap between the two seed sets, with 84 drug-gene pairs in both, 206 in PharmGKB only, and 326 in DrugBank only, and 2898 pairs that were unknown to both.

**Fig 5:**
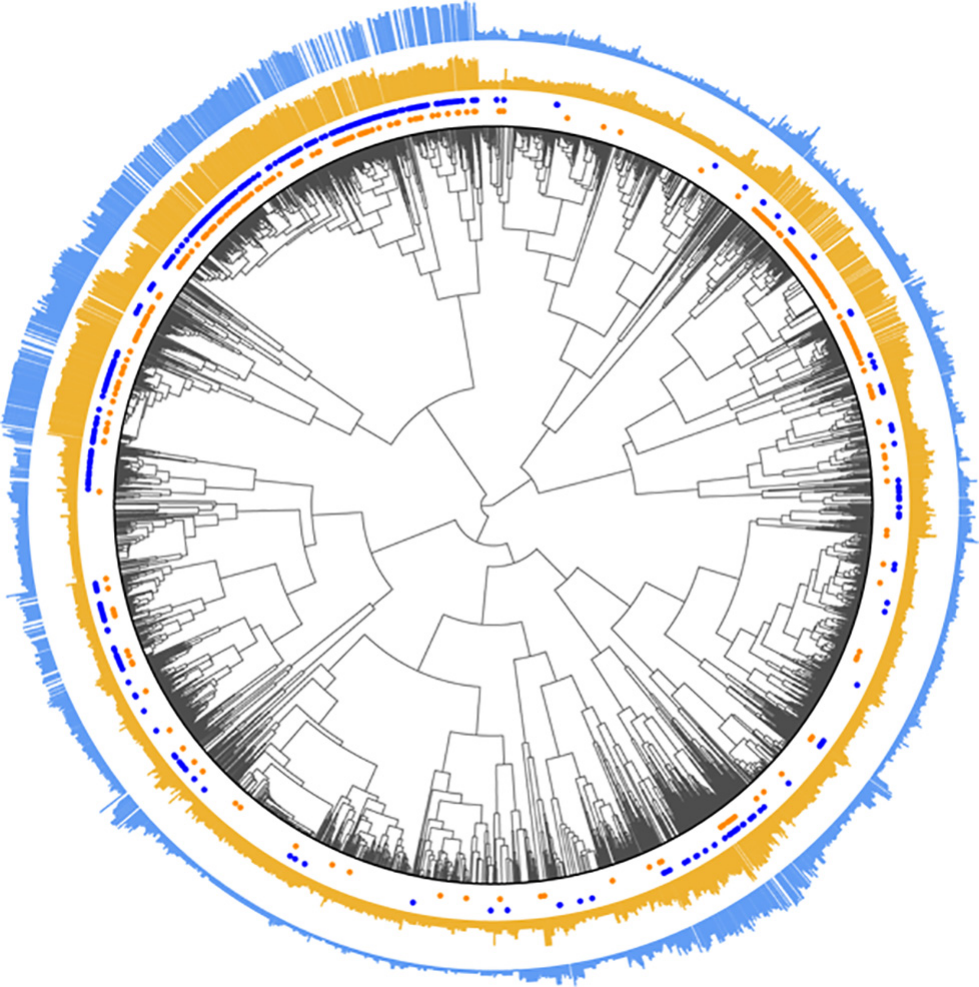
Dendrogram illustrating predictions of novel PGx and drug-target relationships among 3514 drug-gene pairs. The height of the bars corresponds to EBC's certainty that the pair in question represents a relationship of the corresponding type (orange: PGx relationships, blue: drug-target relationships). The dots represent known PGx and drug-target relationships, as in Fig 4.

The dendrogram shown in Fig 5 is identical to that in Fig 4, except that the clusters are replaced by vertical bars, the heights of which correspond to EBC's relative certainty that the pairs in question represent PGx relationships (shown in orange) or drug-target relationships (shown in blue). The raw prediction data can be found in S4 Data. Known PGx or drug-target pairs are excluded from the bar graphs, but are denoted beneath the bars with orange or blue dots. As expected, we see high prediction certainty for drug-target and PGx relationships among the inhibitors in cluster 8, and high certainty for PGx relationships among the metabolic/substrate relationships in cluster 3. We also observe an interesting area of high enrichment for both types of relationships among clusters 21–23, where inhibition is mostly reported in an experimental context, but the density of known PGx and drug-target relationships is quite low. These could represent new experimental findings that will be discussed as static knowledge in a few years.

Table 5 shows the top 20 predictions of new PGx candidate pairs for PharmGKB, and Table 6 shows the top 20 candidate drug-target pairs for DrugBank. Among the top 20 PGx predictions, five are already known to PharmGKB and have been demonstrated experimentally (one or more variants of the gene have been shown to impact response to the drug), but were coded in the PharmGKB relationships file in such a way that they were not included in the seed set. One is brand new: polymorphisms in ABCB1 (P-glycoprotein) do impact clinical response to fentanyl, but this relationship is currently unknown to PharmGKB. An additional eight pairs represent likely PGx relationships, such as known inhibitory or metabolic relationships, but no experiments have yet been conducted that might relate polymorphisms in the gene to drug response. And finally, in five cases, the potential for a PGx association was considered likely enough that it was investigated experimentally, but no significant clinical association between genotype and drug response was found.

**Table 5:** Top 20 predictions of new drug-gene relationships for PharmGKB, and whether a PGx relationship has been documented in the literature.

	Candidate drug-gene pair	Relative certainty	Literature reference (PMID)		Comment
**[1]**	omeprazole, CYP2C19	1.000	11069321	***	Individual polymorphisms of CYP2C19 already associated with omeprazole in PharmGKB.
**[2]**	mexiletine, CYP1A2	0.995	9690950	**	
**[3]**	fentanyl, P-gp	0.994	17192767	***	
**[4]**	voriconazole, CYP3A4	0.986	17433262	**	
**[5]**	cyclosporine, CYP3A4	0.983	18978522	***	Association listed in PharmGKB as “ambiguous”.
**[6]**	duloxetine, CYP1A2	0.983	18307373	**	
**[7]**	fluconazole, UGT2B7	0.982	16542204	**	
**[8]**	montelukast, CYP2C8	0.973	21838784	**	
**[9]**	dydrogesterone, AKR1C1	0.968	20727920	**	
**[10]**	voriconazole, CYP2C9	0.966	16940139	*	
**[11]**	imipramine, FMO1	0.962	19262426	***	Experiment conducted in mice.
**[12]**	ticlopidine, CYP2C19	0.961	21178986	*	
**[13]**	moclobemide, MAO-B	0.960	7586937		In this article, MAO-B activity was studied in relation to moclobemide response, but specific polymorphisms were not investigated.
**[14]**	ritonavir, P-gp	0.958	16184031	***	Association listed in PharmGKB as “ambiguous”.
**[15]**	cyclosporin, MDR1	0.955	15116055	*	
**[16]**	cyclosporin, P-gp	0.952	15116055	*	Same gene as 15.
**[17]**	vinblastine, P-gp	0.951	16917872	***	Association listed in PharmGKB as “ambiguous”.
**[18]**	amprenavir, CYP3A4	0.950	9649346	**	
**[19]**	perazine, CYP1A2	0.945	11026737	**	
**[20]**	lopinavir, ABCB1	0.939	21743379	*	

*** indicates that an association has been demonstrated experimentally between changes in the expression/activity of the gene/protein and the efficacy of the drug

** indicates that such an association is likely, but has not yet been studied

* indicates that the association has been studied experimentally, and the experiment refuted the association. Here we include only associations between pharmaceutical compounds and single genes; predicted associations involving endogenous compounds and/or groups of genes are included in the supplement, however.

**Table 6:** Top 20 predictions of new drug-target relationships for DrugBank.

	Candidate drug-gene pair	Relative certainty	Literature reference (PMID)		Comment
**[1]**	ketanserin, 5-HT2A	1.000	16615363	***	Ketanserin not in DrugBank.
**[2]**	losartan, A-II	0.998	24807206	**	“A-II” refers to the angiotensin type II receptor. In DrugBank this is listed as “Type-1 angiotensin II receptor”.
**[3]**	cangrelor, P2Y12	0.993	20048234	***	Cangrelor not in DrugBank.
**[4]**	phencyclidine, nAChR	0.992	9862757	***	Phencyclidine is a noncompetitive inhibitor of nAChR.
**[5]**	anakinra, IL-1	0.991		*P*	
**[6]**	bosentan, endothelin-1	0.987		*P*	
**[7]**	imatinib, EGFR	0.985	15887238	*	Imatinib's effect on EGFR is ambiguous. It is not likely to be a direct target.
**[8]**	propanolol, Beta2	0.984		*P*	
**[9]**	carvedilol, Alpha1	0.984		*P*	
**[10]**	MK-571, leukotriene	0.983		*L*	MK-571 is unknown to DrugBank.
**[11]**	zafirlukast, leukotriene	0.981		*L*	
**[12]**	degarelix, GnRH	0.980		**	GnRH receptor listed in DrugBank as “Gonadotropin-releasing hormone receptor”. Complicated because degarelix often referred to as “GnRH antagonist” but the target is actually the GnRH *receptor*.
**[13]**	nutlin-3, Mdm2	0.980	18646312	***	Nutlin-3 disrupts the p53-Mdm2 complex. Nutlin-3 is unknown to DrugBank.
**[14]**	genistein, EGFR	0.979	21603581	***	Interestingly, authors found that genistein promotes cancer progression and increases EGFR signaling.
**[15]**	montelukast, leukotriene	0.977		*L*	
**[16]**	aprepitant, NK-1	0.977		**	NK-1 listed in DrugBank as “Substance-P receptor”.
**[17]**	staurosporine, calmodulin	0.975	1846174	*	Staurosporine inhibits calmodulin-dependent protein kinase, not calmodulin.
**[18]**	nutlin-3, Hdm2	0.975	19696166	***	Nutlin-3 is unknown to DrugBank. Hdm2 refers to the human version of the Mdm2 protein (13, above).
**[19]**	tropisetron, 5-HT4	0.974	11243577	***	Tropisetron is unknown to DrugBank.
**[20]**	basiliximab, CD25	0.972	12591363	**	CD25 is listed in DrugBank as “Interleukin-2 receptor subunit alpha”.

*** indicates that the drug has been shown experimentally to have modified the activity of the gene/protein

** means that the interaction is known to DrugBank but is listed under an alternate drug or gene name

* means the interaction has been studied and is unlikely; P refers to a particular type of parser error in which the ligand of a receptor is mistaken for that receptor; L refers to a lexicon error (see Discussion).

Among the top 20 predictions for new drug-target relationships for DrugBank, four are already known but were listed in DrugBank under alternate gene names. An additional seven are new, proven drug-target relationships. Of these, five involve drugs that are themselves unknown to DrugBank (there are no entries for ketanserin, cangrelor, nutlin-3, or tropisetron in DrugBank). There are also several interesting, yet erroneous findings arising from parser and lexicon errors in which a molecule, such as IL-1, is mistaken for its receptor, and that receptor is the true target of the drug. These are explored further in the Discussion.

## Discussion

### Relationship extraction in the biomedical domain

Although a great deal of research effort has been directed at the problem of relationship extraction in pharmacogenomics [17–19], and in the biomedical domain in general [20–25], high-quality biomedical knowledge bases like OMIM, DrugBank and PharmGKB still rely almost entirely on human curators, who comb the literature manually in search of new relationships. The authors of BioGraph, a new biomedical knowledge base incorporating data from 21 different sources, recently decided to exclude databases that were not manually curated, citing data quality issues [26]. Why is biomedical relationship extraction so challenging?

We believe that one key stumbling block lies in how the problem has historically been defined. Biomedical relationship extraction is usually thought of as a sentence-level problem–does a particular sentence describe a specific type of relationship or not? However, as we have seen, sentence-level descriptions are highly erratic. Faced with a bewildering array of possibilities for how similar relationships can be described, sentence-level relationship extraction algorithms often rely on manually-constructed rules or ontologies that map diverse surface forms onto common semantics [17, 27–29]. These systems require a non-trivial amount of human maintenance and must be rebuilt for each new domain. Machine learning algorithms for sentence-level relationship extraction avoid rules but face another serious problem: the need for annotated training sentences. Recently, researchers have begun to produce annotated training sets for the biomedical domain [30, 31] but manual annotation is almost as expensive as manual curation, both in time and human effort. As a result, little to no annotated training data exist for many classes of biomedically interesting relationships.

These are important problems for NLP, but they only exist because we think of biomedical relationships at the level of individual sentences. From a biomedical research standpoint, there is no need to do so—we are most interested in the true relationship between a drug and a gene, not in the meaning of any particular sentence. As a result, we have taken a corpus-level approach where all of the information about a drug-gene pair from all of its available sentence-level descriptions is combined. Latent connections among different-looking descriptions are then discovered in an unsupervised fashion from structure inherent in the raw text, requiring no human effort and boosting our ability to extract relationships of interest.

### Support for corpus-level inference

We contend that biomedical relationships should be considered properties of biomedical entities like drug-gene pairs, not individual sentences. A description like “*D* decreased *G* levels” does not constitute an inhibitory relationship; it is simply an experimental finding that increases the likelihood of such a relationship. This allows the same sentence to provide evidence for or against multiple types of relationship, the exact definitions of which are application dependent. It also allows drug-gene pairs to exhibit multiple relationship types at once.

We see evidence for such an approach when we contrast EBC’s performance at extracting PGx relationships with its performance extracting drug-target relationships. EBC was uniformly worse at extracting PGx relationships, even though these two sets of experiments used the same data matrices. We see why in Fig 4: it turns out that what we originally considered to be well-defined relationship classes (PGx and drug-target relationships) are actually composites of several finer-grained sub-classes. A high percentage of PGx relationships reside in cluster 3, the metabolism/substrate cluster, which inhabits a region of the dendrogram far from the inhibition clusters. In cases where the seed set consists mostly of metabolic relationships and the test set mostly of inhibition relationships, we would not expect EBC to perform well, even though both groups are still technically PGx relationships.

We initially believed that PGx relationships would be expressed in sentences relating specific polymorphisms to changes in drug efficacy, such as, “The CYP3A4 C3435T polymorphism influences rifampicin exposure in human hepatocytes”. In reality, however, relatively few such sentences exist. Most evidence for PGx relationships comes instead from descriptions of other types of relationships, such as inhibition and metabolism. So we see that although a PGx relationship can be considered a property of a drug-gene pair, it is not generally a property of any particular sentence describing that pair.

### Distributional semantics for relationship extraction

EBC is part of a subfield of NLP called distributional semantics, in which patterns in large, unlabeled text corpora are used to create feature representations of words, phrases, or other entities (in our case, drug-gene pairs) based on how they are used in context. The similarity of these representations then serves as a proxy for semantic relatedness [12]. Distributional semantics algorithms’ theme of discovering semantic relatedness by looking at large-scale usage patterns inspired our corpus-level approach to drug-gene relationship extraction. For example, in EBC, these representations are the co-clustering frequencies of each drug-gene pair with every other pair, and the contextual features are the dependency paths.

EBC builds on a long history of distributional semantics work in the NLP literature, much of which focuses on assessing the semantic similarity of individual words [12, 13, 32], and some of which has tackled relationship extraction outside the biomedical domain [33–36]. EBC is most similar in spirit to matrix factorization techniques like Latent Semantic Analysis (LSA) [13]; ITCC forms a low-rank approximation of the original drug-gene-pair-by-dependency-path matrix, and EBC stacks thousands of slightly different ITCC-based approximations on top of each other to make its similarity assessments. LSA uses the singular value decomposition (SVD) [37] instead of ITCC to accomplish a similar goal, and has been applied in at least one case to corpus-level relationship extraction (a technique called Latent Relational Analysis, or LRA) [36]. We compare EBC to LSA on the PGx relationship extraction task in S2 Text.

There are dozens of other clustering and matrix factorization methods available, and some have already been applied to text mining tasks like relationship extraction. Several methods cluster textual patterns to discover latent groupings of entity pairs corresponding to distinct relations [38–41]. Others use the entity pairs flanking different textual patterns to group the patterns themselves into semantically related classes [33]. Some methods, like EBC, address both problems simultaneously [42–45]. The issue of textual “entailment”–finding the degree to which one statement implies the existence of another–is also an active area of research in NLP and is closely related to several of the methods described above [46]. Although these techniques have already shown great promise on related tasks in web and newswire data, to our knowledge none has yet been applied to relationship extraction in the biomedical domain.

### Study limitations: Dependency paths, lexicons and abstracts

In our analysis of drug-gene relationships, we made several choices about (a) how to identify drugs and genes in text, (b) the type of text to use as our corpus, and (c) what constitutes a “feature” (a single column in the data matrix). In all cases, we made the simplest choices possible, both to enable others to reproduce our results, and to distinguish EBC’s own limitations from errors/omissions in the preprocessing steps and text itself.

We identify drugs and genes in the text based on simple string matching to single-word drug and gene names from PharmGKB [5]. Named entity recognition (NER) is its own area of NLP, and identifying biomedical entity names in text is itself a nontrivial proposition. We can see one obvious disadvantage of this approach in cluster 24 of Fig 4 and Table 4, which includes “gene names” like COPD (a.k.a. chronic obstructive pulmonary disease) and NIDDM (non-insulin-dependent diabetes mellitus). Table 6 also reflects a lexicon error where the term “leukotriene” is listed as a synonym for the leukotriene B4 receptor. Some such errors might be avoided if we used a more elaborate NER system [47, 48], though such systems themselves are not perfect and can introduce new sources of error. Our stipulation that the entity names be single words also led to errors in cases (see Table 6) where a molecule, such as IL-1, is mistaken for its receptor, the “IL-1 receptor”, because “IL-1 receptor” is a multi-word phrase not allowed in the lexicon, while “IL-1” is allowed.

We also made no attempt to normalize gene names, so in our results, ABCB1, MDR-1, and P-gp are all different. Again, this was done to avoid introducing normalization errors, and because genes and their corresponding proteins are often described in different contexts.

To construct dependency paths from raw Medline sentences, we used the Stanford Parser [49], a free and open-source statistical parser. The Stanford Parser was trained using labeled text from newswire corpora, so it sometimes fares poorly on biomedical text. For example, the parser often mistakes gene names for adjectives (“CYP3A4” in the phrase “CYP3A4 polymorphism” is frequently labeled as an adjective). We used the out-of-box implementation of the Stanford Parser and did not perform any manual review or correction of parses to improve its performance (again in the interest of simplicity). Because EBC operates at the level of drug-gene pairs and not individual sentences, its performance is generally robust to parsing errors as long as the parser makes the same errors consistently.

There are some errors that do lead to incorrect conclusions, however. For example, we observe some situations where dependency paths bypass important details about relationships, such as a sentence where a drug is described as “transcriptionally up-regulating *G* expression” and the dependency path only captures the effect on expression, not its directionality. These are usually generalizations rather than errors, but they do result in some loss of information from the sentence.

Finally, our corpus consisted of all abstracts from the 2013 edition of Medline. Including information from the full text of the research articles could help discover relationships not mentioned in the abstracts, but many journals do not provide access to the full text, and we did not wish to bias our results in favor of relationships reported in a subset of journals. Our approach would remain the same regardless of the corpus.

### Extensions and future applications

The combination of EBC and dependency path features described here allows us to reliably extract biomedical relationships of interest from Medline sentences, smoothing over differences in how these relationships are described. This finding opens the door to many interesting possible future applications. For example, EBC could be used to extract relationships spanning multiple sentences or entire abstracts by using features such as individual dependencies, words, or phrases in place of dependency paths. As new gold-standard sets of biomedical relationships become available (such as all drug-gene pairs reflecting inhibitory relationships or specific collections of drug-gene pairs relevant to particular laboratories’ research efforts) these can seamlessly be incorporated into EBC to extract these relationships at scale. EBC could also potentially be used for lexicon or ontology expansion in a manner similar to LSA or random indexing [50, 51]. At its core, EBC is not relationship extraction-centric. The algorithm itself is agnostic to the type of data contained in its input matrix. EBC simply allows us to use latent structure in large, unlabeled datasets to boost our ability to extract new information from those datasets, even when our access to labeled training examples is limited. Datasets like these occur throughout biomedical research, even beyond NLP. We look forward to seeing how EBC fares on some other classes of related problems, in NLP and elsewhere.

## Methods

### Outline of the EBC algorithm

When applied to drug-gene relationship discovery, the EBC algorithm operates on a data matrix where the rows are drug-gene pairs and the columns are dependency paths that connect them in the literature. The algorithm has two steps, the first unsupervised and the second supervised.

First, unsupervised biclustering is used to simultaneously discover (a) latent connections among dependency paths (columns) that appear different but connect similar drug-gene pairs, and (b) latent similarities among different drug-gene pairs (rows) that are connected by similar dependency paths. Over multiple iterations of (a) and (b), the algorithm can infer that two drug-gene pairs share a similar relationship, even when they share no dependency paths in common. To make its similarity assessments, EBC uses an ensemble of biclustering runs where the cluster centers are initialized randomly on each run, providing many different guesses about which dependency paths and drug-gene pairs are related.

In the second step, EBC incorporates a small seed set of drug-gene pairs (rows) reflecting some known relationship, and ranks other pairs based on their similarity to the pairs in the seed set. The specific steps of the EBC algorithm are as follows:

Preprocessing (drug-gene relationship extraction task):

1. Identify all drug-gene pairs co-occurring in sentences within a corpus of text. (In our experiments, these were drug-gene pairs co-occurring in Medline sentences.) Call the number of drug-gene pairs *n*.
2. Extract all dependency paths connecting these drug-gene pairs in the corpus. Call the total number of observed paths *m*.
3. Arrange the data in an *n* x *m* matrix where the rows represent drug-gene pairs and the columns dependency paths. A cell with coordinates *(i*, *j)* in this matrix contains “1” if drug-gene pair *i* has been connected by path *j* somewhere in the corpus, and “0” otherwise.

EBC algorithm:

4. (Unsupervised step.) Use Information-Theoretic Co-Clustering (ITCC; [16], details below) to bicluster the *n* x *m* matrix *N* times, recording the number of runs in which each row appears in a row cluster with each other row. The result is an *n* x *n* array, *C*, of co-occurrence values. Note that no information about the seed set is incorporated at this stage, so the unsupervised step need be run only once per data matrix.
5. (Supervised step.) Identify a seed set, *S*, of rows that share some property of interest. (In our experiments, these were drug-gene pairs with known PGx or drug-target relationships.) Rank the entity pairs in a test set, *T*, based on a scoring function related to how often they co-cluster with members of *S* (details below). Repeat this step as desired with different seed sets.

### Named entity recognition of drugs and genes

We identified drug and gene entity names in the text using simple string matching to lexicons, though any type of named entity recognition software could be incorporated at this stage [47, 48]. We obtained drug and gene lexicons from PharmGKB [5] and filtered them against a dictionary of common English words to remove promiscuous terms (such as “CAT”, which is both a gene name and an animal). We included only drug and gene entities with one-word names, as these names mapped to single nodes in the dependency graphs. The final drug lexicon contained 4008 unique terms, and the final gene lexicon contained 109,597 terms (many genes/proteins had multiple names).

### Extraction of dependency paths from Medline abstracts

We used the Stanford Parser [49] to generate dependency graphs for all sentences in Medline 2013 between 4 and 50 words in length (roughly 95% of all sentences in Medline). The input to the parser is a raw Medline sentence, and the output is a dependency graph. A dependency graph (see Fig 1) is one way to represent the grammatical architecture of a sentence; the nodes are words, and the edges are grammatical dependencies (grammatical relationships between pairs of words, described in detail in [15]).

A dependency path is a path through a dependency graph that connects two entities of interest. Considering a dependency path, instead of an entire sentence, can help “prune out” irrelevant terms and phrases and focus our attention on the part of the sentence directly relevant to the relationship between the two entities. We extracted all dependency paths linking drugs to genes.

It was possible for a single sentence to generate more than one dependency path if multiple drug or gene names were present in the sentence. We oriented our paths so that they always started at the drug and ended at the gene, and we eliminated edge directions. (We never observed a single situation where we accidentally collapsed paths with different meanings in doing so, since most pairs of words can only be connected by a particular dependency type, like *amod* or *nn*, in one direction.) We eliminated paths containing dependencies of type *conj* [15], because these were usually errors arising from inadequacies in how the dependency parser represents lists. Note that because the dependency graphs are trees, there is one unique dependency path for each drug-gene pair in a sentence.

### Ensemble biclustering

ITCC forms a low-rank approximation of a matrix by iteratively clustering the rows and columns. ITCC treats the data matrix, *M*, as a joint probability distribution over its rows (*Y*, drug-gene pairs) and columns (*X*, dependency paths). Given fixed numbers of row (*k*) and column (*l*) clusters, ITCC finds a set of cluster assignments for the rows and columns that captures most of the mutual information between *X* and *Y* with the stipulation that *X* and *Y* only interact via their cluster assignments, $\hat{X}$ and $\hat{Y}$. Mathematically, ITCC replaces the joint distribution of *X* and *Y*, $p(x,y)=p(\hat{x},\hat{y})\;p(x,y|\hat{x},\hat{y})$, with an approximate distribution of the form $q(x,y)=q(\hat{x},\hat{y})\;q(x|\hat{x})q(y|\hat{y})$, and assigns rows and columns to clusters so that *q*(*x*,*y*) captures most of the mutual information between *X* and *Y* in *p*(*x*,*y*) (equivalent definition: the Kullback-Leibler divergence between *p*(*x*,*y*) and *q*(*x*,*y*) is minimized). We implemented ITCC in Java. Some technical details about our implementation can be found in S3 Text.

There are two unknown input parameters to ITCC: the numbers of row (*k*) and column (*l*) clusters. The optimal choices for *k* and *l* must be decided heuristically. We describe our heuristic for choosing *k* and *l* in S1 Text A.

Due to random initialization of the row and column cluster centers, ITCC generally converges to a different locally-optimal biclustering on each run; this diversity is what guarantees optimal performance of the EBC algorithm. We ran ITCC *N* = 2000 times at the optimal *k* and *l* and recorded the number of runs in which each pair of rows shared a cluster. We observed that on our data matrices, EBC’s performance increased monotonically with *N*, stabilizing at approximately *N* = 1000.

### Scoring of test set pairs

Once EBC’s unsupervised step is performed and appropriate seed (*S*) and test (*T*) sets identified, test set items can be ranked as follows:

1. **EBC’s scoring function.** For each test set member, *T*_*i*_, rank all *n* rows of the data matrix based on how often they co-cluster with *T*_*i*_. This produces a ranking *R*_*i*_ of length *n* in which pairs that frequently co-cluster with *T*_*i*_ are assigned high ranks and those that seldom co-cluster get low ranks. The score for *T*_*i*_ is the rank sum of the members of the seed set, *S*, within this list, or:
  $$\text{score}(T_{i})=\sum_{j=1}^{n}j∙I\{R_{ij}\in S\}$$
  where
  $$I\{R_{ij}\in S\}=\left\{ \begin{array}{ll} \;1 & \text{if}\;R_{ij}\in S\\ \;0 & \text{otherwise} \end{array}\right.$$
  Using ranks instead of absolute co-clustering frequencies produces a score that does not depend on how often, on average, a given drug-gene pair co-clusters with other pairs, since this baseline “promiscuity” changes from pair to pair. For some applications, those differences might not matter (or they might be informative) but we normalized to ranks so promiscuous pairs (which are often well-known or frequently mentioned pairs) would not consistently receive higher scores than less promiscuous pairs. EBC’s scoring function will assign a high score to a test set member as long as the seed set rows tend to cluster with it more frequently than other rows do. Ties are broken randomly.
  We compared EBC’s performance to two other ranking methods that did not take the semantic similarity of different dependency paths into account:
2. **AvgCosine.** Let $v_{T_{i}}$ be the row vector in the data matrix associated with test set member *i*. This vector contains *m* elements: one for each dependency path. Let $v_{S_{j}}$ be the row vector associated with seed set member *j*. Here we score each test pair *T*_*i*_ based on the average cosine similarity of $v_{T_{i}}$ with all of the row vectors from the seed set, or:
  $$\text{score}(T_{i})=\frac{1}{\left|S\right|}\sum_{j=1}^{\left|S\right|}\frac{v_{T_{i}}\cdot v_{S_{j}}}{\left\|v_{T_{i}}\|\|v_{S_{j}}\right\|}$$
  where ‖⋅‖ denotes the Euclidean norm.
3. **RankSum.** In keeping with the spirit of EBC’s scoring function, for each *T*_*i*_ we rank all *n* rows of the data matrix based on cosine similarity to $v_{T_{i}}$. This produces a ranking *R*_*i*_ of length *n* in which rows with high cosine similarity to $v_{T_{i}}$ are assigned high ranks and those with low cosine similarity to $v_{T_{i}}$ get low ranks. The score for *T*_*i*_ is the rank sum of the members of *S* within this list, and looks identical to that for EBC; the only difference is that the rankings *R*_*i*_ are produced using cosine similarity and not EBC.

### Evaluating rankings of PGx and drug-target relationships

For both the PGx and drug-target tasks, and for seed set sizes |*S*| = 1, 2, 3, 4, 5, 10, 25, 50, and 100, we generated 1000 random seed sets and 1000 corresponding test sets, ensuring that the seed sets and test sets did not overlap. The test sets were all composed of 100 drug-gene pairs, 50 of which had known PGx or drug-target relationships and 50 of which did not. All three ranking methods were used to rank the members of each test set, using its associated seed set for scoring.

We also explored the impact of data sparsity by performing these evaluations on two separate datasets. In the “dense” dataset, we included only drug-gene pairs and dependency paths that occurred at least five times in Medline. In the “sparse” dataset, we included dependency paths occurring at least twice, and any drug-gene pairs they connected (even if they only co-occurred in a single sentence). More information about the two datasets can be found in Table 2, and the data matrices themselves can be found in S2 Data.

We evaluated the quality of each ranking by calculating the area under the receiver operating characteristic curve (AUC) [52], a measure of how likely it is that a positive element of the test set will be ranked higher than a negative element. We elected to use AUC instead of precision or recall because we wanted a threshold-independent measure of the overall quality of the ranking. We used R’s ROCR package to calculate the AUCs. From a practical standpoint, we were concerned mainly with the following scenario: Given that I have a seed set about whose quality I know nothing, what is the chance I can accurately prioritize the knowledge I am looking for within my [unlabeled] corpus? Our evaluation metric was, therefore, the fraction of the 1000 seed sets that ranked their corresponding test sets with AUC > 0.7.

### Comparing EBC to Latent Semantic Analysis (LSA)

To investigate how similar EBC’s performance was to a more established method designed to solve a similar problem, we used the singular value decomposition (SVD) [37] to decompose our two data matrices, creating “compressed” feature vectors of reduced dimensionality for each drug-gene pair and incorporating these, rather than the raw row vectors, into the two non-EBC ranking methods described above. This approach is identical to the famous text mining technique Latent Semantic Analysis (LSA; [13]) which was originally applied to overcome issues of data sparsity in document retrieval. The results of these experiments are described further in S2 Text.

### Building a dendrogram of drug-gene pairs based on EBC’s similarity assessments

EBC provides a natural measure of similarity for each drug-gene pair and every other pair: the number of times the rows corresponding to those two pairs clustered together over the *N* biclustering runs. However, as we have seen, these raw values are not fair measures of distance for all pairs, since some drug-gene pairs tend to cluster frequently with many other pairs, and others cluster less frequently. EBC’s rank-based scoring function accounts for this by normalizing to ranks: each drug-gene pair ranks all other pairs by co-clustering frequency, and these ranks are used in place of the raw co-clustering values in the scoring function.

To implement EBC's scoring function in an unsupervised manner to construct our dendrogram, we started with our *n* x *n* matrix of co-occurrence values, *C*, in which *C*_*ij*_ was the number of runs (out of *N* total) in which drug-gene pair *i* co-clustered with drug-gene pair *j*. We then converted *C* into a correlation matrix, *ρ*, also *n* x *n*, where *ρ*_*ij*_ contained the Spearman correlation of *C*_*i*⋅_ and *C*_*j*⋅_, the *i*th and *j*th rows of *C* (note that *C* is symmetric, so we could just as easily have used columns). These correlations are, as in EBC's scoring function, measures of how similarly drug-gene pair *i* and pair *j* rank all other pairs in the matrix, and are not biased in favor of promiscuous pairs. We then used 1 − *ρ* as the distance measure for hierarchical clustering using minimax linkage [53] to produce the dendrogram shown in Fig 4. Using a different linkage function or distance metric, obviously, would produce a different-looking dendrogram.

We used several R packages to produce the dendrogram figures, including *ape* (a library for making phylogenetic trees), and *protoclust* (a library for hierarchical clustering using minimax linkage). To achieve the radially-spaced tip markers, we used a separate package [54].

## Supporting Information

S1 Text Optimizing row and column cluster numbers for EBC. We describe our heuristic for choosing the optimal number of row (*k*) and column (*l*) clusters for EBC based on the structure of the data matrix.
(PDF)

S2 Text Comparing EBC to Latent Semantic Analysis (LSA). We compare EBC to another related technique that was one of the first to use matrix decompositions to address the problem of data sparsity in text mining.
(PDF)

S3 Text Technical details about our implementation of EBC in Java. (PDF)

S1 Data Co-clustering frequencies on dense and sparse matrices. We provide the raw co-clustering frequencies of the rows (drug-gene pairs) of both matrices over *N* = 2000 runs.
(PDF)

S2 Data Sparse and dense data matrices for the drug-gene relationship extraction task, stored in a sparse format. (PDF)

S3 Data Cluster assignments for the dendrogram in Fig 4, at five different cut heights. (PDF)

S4 Data Prediction certainties from Fig 5 for PharmGKB and DrugBank. (PDF)
