# Supplementary material for: Learning the Structure of Biomedical Relationships from Unstructured Text
Source: PLoS Comput Biol. 2015 Jul 28;11(7):e1004216. doi: 10.1371/journal.pcbi.1004216 (PMC4517797; doi:10.1371/journal.pcbi.1004216)
Supplement: S1 Text — We describe our heuristic for choosing the optimal number of row (k) and column (l) clusters for EBC based on the structure of the data matrix. (PDF) [file pcbi.1004216.s001.pdf]

SUPPLEMENT A: OPTIMIZING ROW AND COLUMN CLUSTER NUMBERS FOR EBC

The EBC heuristic for optimizing cluster numbers

There are two unknown parameters that ITCC requires as input: the numbers of row (k) and column (ℓ) clusters. The objective function of the ITCC algorithm is the difference in the mutual information between X and Y in the original dataset, $I(X,Y)$, and between the clusters $\hat{X}$ and $\hat{Y}$ in the clustered dataset, $I(\hat{X},\hat{Y})$, or $I(X,Y) - I(\hat{X},\hat{Y})$. This can also be expressed as the Kullback-Leibler divergence between the original joint distribution of X and Y, $p(x,y) = p(\hat{x},\hat{y})p(x,y|\hat{x},\hat{y})$, and the approximate distribution produced by ITCC, $q(x,y) = q(\hat{x},\hat{y})q(x|\hat{x})q(y|\hat{y})$. This objective generally decreases as k and ℓ increase, since by introducing more clusters, the approximate distribution $q(x,y)$ more closely approximates the original distribution $p(x,y)$. Therefore, we need a separate heuristic to decide on the optimal k and ℓ.

The key fact that led us to our solution is that the objective function decreases with increasing k and ℓ no matter what the original matrix (M) looks like. In particular, this should be true if we choose any random matrix (M_r) whose $n \times m$ individual elements are the same as those of M, but where the locations of those elements within the matrix are randomized with respect to both rows and columns. We can think of the "optimal clustering" as one that captures the structure inherent in M using as few clusters as possible. We therefore reasoned that we should search for k and ℓ such that the value of the objective function for the clustering over M was as low as possible relative to its value for M_r, meaning that the clustering captured more of the original mutual information $I(X,Y)$ than would be expected due to chance. In other words, we sought to minimize

$$\text{objective}(M) - \text{objective}(M_r) \tag{1}$$

over many different randomized M_r matrices. We performed grid searches over ranges of k and ℓ from 1 to 400 (100 separate clusterings at each (k,ℓ); grid size = 5) for both data matrices and identified the (k,ℓ) that minimized Equation 1. For the sparse matrix, we performed a finer-grained grid search over the row cluster numbers, since the optimal value on the first search was 5 – this is what allowed us to arrive at our final value of $k = 7$ for that matrix.

In spirit, our heuristic most resembles the popular gap statistic for unidimensional clustering [1].

Examples of EBC heuristic on small matrices

We present here three examples of small matrices, and show how the EBC heuristic can be used to find k and ℓ for each matrix.

Example 1 This is the original example matrix used in Dhillon *et al*'s 2003 paper [2]. In the paper, the authors choose $k = 3$ as the number of row clusters and $\ell = 2$ as the number of column clusters, but our heuristic finds $k = 4$ and $\ell = 3$. Beside the matrix is the contour plot for the optimization of

the score given in Equation 1. In this plot, dark denotes a low value of Equation 1 (good) and light denotes a high value (bad).

**0.05**	**0.05**	**0.05**	0.00	0.00	0.00
**0.05**	**0.05**	**0.05**	0.00	0.00	0.00
0.00	0.00	0.00	**0.05**	**0.05**	**0.05**
0.00	0.00	0.00	**0.05**	**0.05**	**0.05**
**0.04**	**0.04**	0.00	**0.04**	**0.04**	**0.04**
**0.04**	**0.04**	**0.04**	0.00	**0.04**	**0.04**

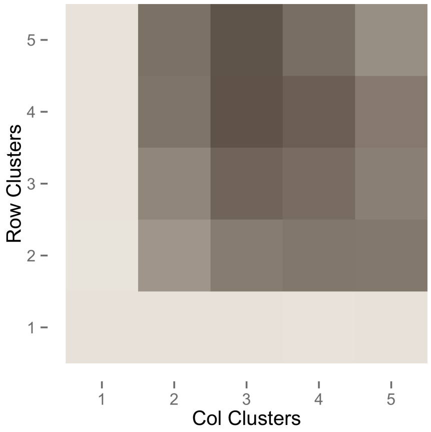

Example 2 Here is an example where the matrix obviously contains two row and two column clusters:

**0.05**	**0.05**	**0.05**	0.00	0.00	0.00
**0.05**	**0.05**	**0.05**	0.00	0.00	0.00
0.00	0.00	0.00	**0.05**	**0.05**	**0.05**
0.00	0.00	0.00	**0.05**	**0.05**	**0.05**
**0.067**	**0.067**	**0.067**	0.00	0.00	0.00
**0.067**	**0.067**	**0.067**	0.00	0.00	0.00

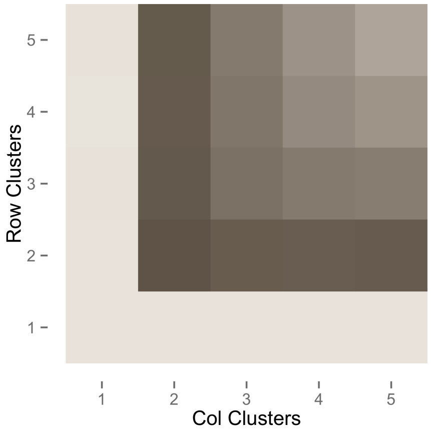

Example 3 Finally, here is an example where the matrix obviously contains three row and three column clusters:

**0.083**	**0.083**	0.00	0.00	0.00	0.00
**0.083**	**0.083**	0.00	0.00	0.00	0.00
0.00	0.00	**0.083**	**0.083**	0.00	0.00
0.00	0.00	**0.083**	**0.083**	0.00	0.00
0.00	0.00	0.00	0.00	**0.083**	**0.083**
0.00	0.00	0.00	0.00	**0.083**	**0.083**

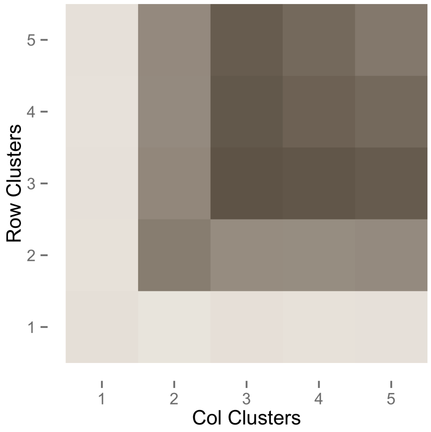

Properties and advantages of this heuristic

When performing the grid searches, we noted that there appeared to be single, connected regions on the (k, ℓ) plane where Equation 1 was minimized, but that the area surrounding the minimum was relatively flat, suggesting that there were probably many different combinations of cluster numbers that would have yielded similar results. We observed that if we varied k and ℓ by ± 10 from their optimal values, performance was effectively unchanged. If we drastically changed k and ℓ (such as setting both to 2), performance declined. In future work, we plan to investigate how sensitive our results are to the choices of k and ℓ. Because the grid search is the most computationally expensive part of our method, it would be ideal if k and ℓ could be chosen from broad ranges without reducing the performance of subsequent classifiers very much.

Using the EBC heuristic means we choose k and ℓ based entirely on the structure of the data matrices, without regard for how these choices will affect the eventual performance of the EBC classifier. It is, therefore, possible that there are other k and ℓ values that would lead to the same or even better performance than we obtained here. We declined to search for them because we valued being able to choose k and ℓ in a task-independent manner.

A note on the cluster numbers chosen for sparse and dense matrices

The optimal numbers of clusters for our two data matrices vary considerably and in non-obvious ways (see Table 3 in the main text), with the larger, sparser dataset requiring fewer clusters than the smaller, denser dataset. It seems that the higher degree of nonzero-element overlap between rows and columns in the dense dataset supports a finer-grained assignment of rows and columns to clusters. This finding highlights the need for a reliable heuristic for selecting cluster numbers.

References

[1] Tibshirani, R., Walther, G., & Hastie, T. (2001). Estimating the number of clusters in a data set via the gap statistic. Journal of the Royal Statistical Society: Series B (Statistical Methodology), 63(2), 411-423.

[2] Dhillon, I. S., Mallela, S., & Modha, D. S. (2003, August). Information-theoretic co-clustering. In Proceedings of the ninth ACM SIGKDD international conference on Knowledge discovery and data mining (pp. 89-98). ACM.
