# Supplementary material for: Learning the Structure of Biomedical Relationships from Unstructured Text
Source: PLoS Comput Biol. 2015 Jul 28;11(7):e1004216. doi: 10.1371/journal.pcbi.1004216 (PMC4517797; doi:10.1371/journal.pcbi.1004216)
Supplement: S2 Text — We compare EBC to another related technique that was one of the first to use matrix decompositions to address the problem of data sparsity in text mining. (PDF) [file pcbi.1004216.s002.pdf]

Supplement B: Comparing EBC to Latent Semantic Analysis (LSA)

To compare EBC's performance to a more established method designed to solve a similar problem, we used the singular value decomposition (SVD) [1] to decompose the sparse and dense data matrices, creating "compressed" feature vectors of reduced dimensionality (of various lengths) for each drug-gene pair and incorporating these, rather than the raw row vectors, into the two non-EBC ranking methods described in the Methods (AvgCosine and RankSum). This approach is virtually identical to the famous text mining technique Latent Semantic Analysis (LSA; [2]), except that our matrices are binary while the original matrices in the LSA paper used counts.

The results of the PGx relationship extraction task on the dense and sparse matrices are below. The RankSum ranking method appeared to work the best for LSA. We include the results for the RankSum method used on the original feature vectors (denoted by "RankSum") and on various lengths of compressed vectors (denoted by their vector lengths). For comparison, we also include the results for EBC.

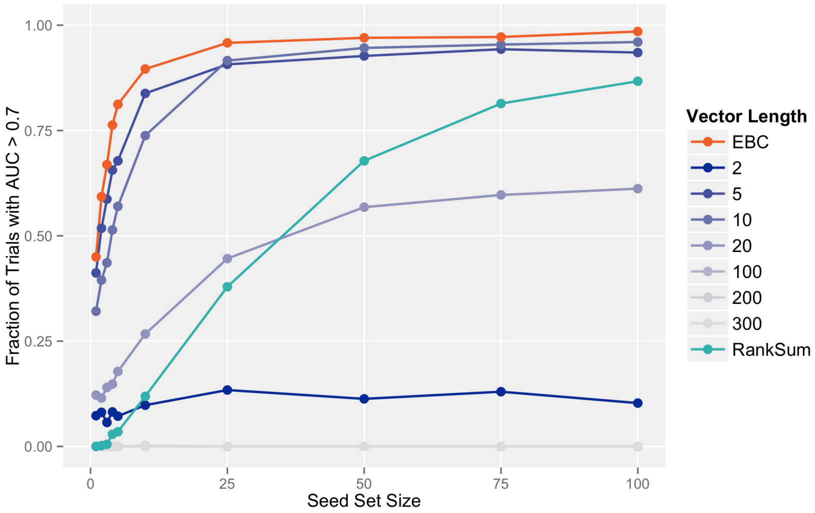

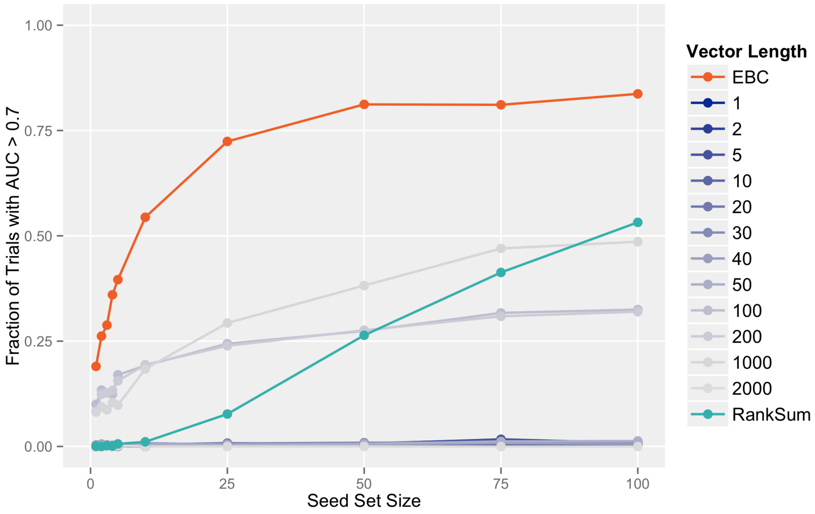

We see that the performance of classifiers that rely on the SVD for dimensionality reduction strongly depends on the length of the compressed feature vectors, and the best-performing vector length varies with the size and structure of the data matrix. For the dense matrix, the optimal vector length was somewhere between 5 and 10 (depending on the size of the seed set), while for the sparse matrix, vectors of length 5 or 10 performed horribly, and the optimal length was somewhere between 1000 and 7272 (the uncompressed vector length). For the dense matrix, if the correct vector length was chosen, the results came close to those of EBC, but for the sparse matrix (unless there was a specific vector length between 1000 and 7272 that led to a rapid increase in performance) the results never approached EBC's.

More importantly, in a real curation scenario, the vector length would need to be chosen using a development set (necessitating additional training data beyond the seed set) or via some other heuristic, and the strong relationship between vector length and classifier performance means we would be unlikely to choose optimal values randomly. The authors of the original LSA paper specifically mentioned the choice of vector length as one of the major challenges facing their algorithm, so the heuristic in Supplement A turns out to be one of EBC's key advantages.

References

[1] Skillicorn, D. (2007). Understanding complex datasets: data mining with matrix decompositions. CRC press.

[2] Deerwester, S. C., Dumais, S. T., Landauer, T. K., Furnas, G. W., & Harshman, R. A. (1990). Indexing by latent semantic analysis. JASIS, 41(6), 391-407.
