# Supplementary material for: Learning the Structure of Biomedical Relationships from Unstructured Text
Source: PLoS Comput Biol. 2015 Jul 28;11(7):e1004216. doi: 10.1371/journal.pcbi.1004216 (PMC4517797; doi:10.1371/journal.pcbi.1004216)
Supplement: S3 Text — (PDF) [file pcbi.1004216.s003.pdf]

## Supplement G: Technical details

### *Why does EBC work?*

The key to EBC's increased performance on our two relationship identification tasks is its ability to implicitly relate different-looking descriptions of similar phenomena. EBC reasons "by analogy". If two drug-gene pairs are connected by similar sets of dependency paths, EBC infers that they are related. Similarly, if two dependency paths connect similar sets of drug-gene pairs, EBC infers that the meaning of those paths is similar. Over several iterations, EBC is able to quantitatively estimate the relatedness of two drug-gene pairs, even when they share no dependency paths in common.

### *Implementation details*

Here are a few final practical notes on implementing EBC:

1. There is no reason why the matrix elements of the data matrices, $M$, could not be continuous values, or integers greater than 1. In our matrices, they were binary (0/1), but in Supplement A, they were numeric values. If one only wants to work with binary matrices, however, it is possible to make EBC faster and less memory intensive by simply storing the locations of the nonzero elements in the matrix and storing the single, nonzero value of these elements as a separate variable.

2. In Dhillon and Modha's original algorithm, it was possible to "lose" clusters over the course of one run of ITCC. This could happen, for example, if two rows were chosen as cluster centers that had exactly the same conditional distributions over the columns. One cluster center row could then be assigned to the other cluster, reducing the total number of row clusters by 1. We therefore introduced a check in our code: after each iteration of ITCC, the code checks to see if the desired numbers of rows and columns are present; if not, it randomly chooses rows/columns from within the existing clusters and relabels them as the cluster centers of the missing clusters. It then checks again to ensure that the total numbers of row and column clusters are correct, and continues the random assignment process until they are. This was especially important for the grid search that determined optimal cluster numbers.

3. To initially assign rows and columns to clusters, we chose the desired numbers of row and column cluster centers from among the rows and columns of the matrix and matched the rest of the rows and columns to clusters based on cosine similarity with their cluster centers. If a row or column had zero cosine similarity with all of the cluster centers (zero overlap with all) it was initialized to the first cluster, so all of the "leftover" rows and columns started out in the same clusters.

### *Key advantages of EBC*

While it is certainly possible that another matrix approximation method could produce results comparable to EBC, EBC does have several advantages that make it particularly well-suited to the

relationship extraction problem (especially when the data are sparse, as shown in the main text). The first is speed. The time complexity of ITCC is $\mathcal{O}(z \cdot \tau \cdot (k + \ell))$, where $z$ is the number of nonzero elements in the data matrix, $\tau$ is the number of iterations the algorithm takes to converge (usually $< 20$) and $k$ and $\ell$ are the numbers of row and column clusters. This gives EBC an advantage over algorithms, like those for computing the SVD, whose runtime depends on powers of both $m$ (total number of columns) and $n$ (total number of rows), especially since the $N$ different ITCC runs for EBC are independent and can easily be parallelized. And finally, the unsupervised step of EBC needs to be run only once per data matrix. Subsequent classification tasks using different seed sets can use the stored ensemble biclustering data and are, therefore, extremely fast.

A second advantage of EBC is that $k$ and $\ell$ are its only tuning parameters, and these can be set using the heuristic described in Supplement A, which assigns them based solely on the structure of the data matrix itself. Many of EBC's sister methods contain parameters like these that need to be optimized to a particular task using a development set of additional labeled training data, but this defeats the purpose of a method designed to work in situations where little to no annotated training data is available. As one can see from Supplement B, for example, LSA's performance is strongly impacted by the dimensionality of the compressed feature vectors, a fact that its authors noted in their original paper as one of its major limitations [1]. Using EBC avoids this problem.

## REFERENCES

[1] Deerwester, S. C., Dumais, S. T., Landauer, T. K., Furnas, G. W., & Harshman, R. A. (1990). Indexing by latent semantic analysis. JASIS, 41(6), 391-407.
