# Supplementary material for: Learning the Structure of Biomedical Relationships from Unstructured Text
Source: PLoS Comput Biol. 2015 Jul 28;11(7):e1004216. doi: 10.1371/journal.pcbi.1004216 (PMC4517797; doi:10.1371/journal.pcbi.1004216)
Supplement: S1 Data — We provide the raw co-clustering frequencies of the rows (drug-gene pairs) of both matrices over N = 2000 runs. (PDF) [file pcbi.1004216.s004.pdf]

Supplement C: Co-clustering frequencies on dense and sparse matrices

Two data files are provided that contain the raw coclustering frequencies of the rows in the sparse and dense data matrices over $N = 2000$ runs. The file format is tab-delimited, and looks like this:

```
(flavopiridol,nf-kappab) 0 (flavopiridol,nf-kappab) 0 2000
(flavopiridol,nf-kappab) 0 (tnf-r2,tnf-r1) 1 36
(flavopiridol,nf-kappab) 0 (il-2,il-5) 2 7
(flavopiridol,nf-kappab) 0 (il-11,il-10) 3 8
(flavopiridol,nf-kappab) 0 (fgf-7,fgf-2) 4 9
(flavopiridol,nf-kappab) 0 (clopidogrel,p-selectin) 5 25
(flavopiridol,nf-kappab) 0 (fgf-7,fgf-1) 6 12
(flavopiridol,nf-kappab) 0 (il-11,il-13) 7 10
(flavopiridol,nf-kappab) 0 (propranolol,beta2) 8 31
(flavopiridol,nf-kappab) 0 (isoflurane,caspase-3) 9 400
(flavopiridol,nf-kappab) 0 (indomethacin,phospholipase) 10 2
(flavopiridol,nf-kappab) 0 (anti-il-2,cd25) 11 59
(flavopiridol,nf-kappab) 0 (zuclopenthixol,cyp2d6) 12 13
(flavopiridol,nf-kappab) 0 (doxycycline,smad7) 13 34
(flavopiridol,nf-kappab) 0 (il-2,il-1) 14 12
(flavopiridol,nf-kappab) 0 (corticosteroids,leukotriene) 15 30
(flavopiridol,nf-kappab) 0 (gliclazide,niddm) 16 16
(flavopiridol,nf-kappab) 0 (tolbutamide,niddm) 17 26
(flavopiridol,nf-kappab) 0 (dipyridamole,pde5) 18 3
(flavopiridol,nf-kappab) 0 (geldanamycin,hsp90) 19 0
```

where the order of the columns is:

```
drug-gene-pair-1 numeric-id-1 drug-gene-pair-2 numeric-id-2 cooccurrence-count
```

The names of the two files are:

`rows-same-cluster-30-125-new.tsv`
(results for dense matrix, $k = 30$, $\ell = 125$)

`rows-same-cluster-fullmatrix-7-25-new.tsv`
(results for sparse matrix, $k = 7$, $\ell = 25$)
