# Supplementary material for: Learning the Structure of Biomedical Relationships from Unstructured Text
Source: PLoS Comput Biol. 2015 Jul 28;11(7):e1004216. doi: 10.1371/journal.pcbi.1004216 (PMC4517797; doi:10.1371/journal.pcbi.1004216)
Supplement: S2 Data — (PDF) [file pcbi.1004216.s005.pdf]

Supplement D: Dense and sparse matrices

Two files are provided that contain the dense and sparse matrices themselves in a sparse format. The file format is tab-delimited. The dense matrix file looks like this:

```
3514
1232
(flavopiridol,nf-kappab) 0 [prep_by, activation, amod] 781 1.0
(tnf-r2,tnf-r1) 1 [appos, receptor, appos] 413 1.0
(tnf-r2,tnf-r1) 1 [pobj, to, dep, to, pobj] 1125 1.0
(tnf-r2,tnf-r1) 1 [amod, expression, prep_of] 1179 1.0
(il-2,il-5) 2 [appos, csf, appos] 24 1.0
(il-2,il-5) 2 [appos, ngf, appos] 38 1.0
(il-2,il-5) 2 [nsubj, induced, acomp] 122 1.0
(il-2,il-5) 2 [dep, factor, dep] 240 1.0
(il-2,il-5) 2 [nsubj, induced, dobj, expression, prep_of] 321 1.0
(il-2,il-5) 2 [appos, rantes, appos] 344 1.0
(il-2,il-5) 2 [dep, alpha, dep] 385 1.0
(il-2,il-5) 2 [appos, alpha, appos] 430 1.0
(il-2,il-5) 2 [prep_with, synergized, nsubj] 431 1.0
(il-2,il-5) 2 [agent, induced, partmod, expression, nn] 595 1.0
(il-2,il-5) 2 [appos, interleukin, appos] 622 1.0
(il-2,il-5) 2 [prep_of, amounts, amod] 633 1.0
(il-2,il-5) 2 [nsubj, induced, dobj, expression, nn] 807 1.0
(il-2,il-5) 2 [appos, lt, appos] 909 1.0
```

where the first row contains the number of rows in the matrix (3514), the second the number of columns (1232) and the rest the nonzero matrix elements in the matrix (there are $10,007$ nonzero elements in the dense matrix and $29,456$ in the sparse matrix). The columns are as follows:

`row-name row-id col-name col-id value`

The element values will always be 1.0 for these matrices, but could take other numeric values for, say, the example matrices in Supplement A. Note that the numeric IDs for these matrix rows do not match the numeric IDs in the cooccurrence files in Supplement C.

The names of the two files are:

`matrix.tsv`
(dense matrix)

`matrix-full.tsv`
(sparse matrix)
