# Supplementary material for: Learning the Structure of Biomedical Relationships from Unstructured Text
Source: PLoS Comput Biol. 2015 Jul 28;11(7):e1004216. doi: 10.1371/journal.pcbi.1004216 (PMC4517797; doi:10.1371/journal.pcbi.1004216)
Supplement: S3 Data — (PDF) [file pcbi.1004216.s006.pdf]

Supplement E: Cluster assignments for the dendrogram in Figure 4

The file `cluster-assignments.tsv` contains the cluster assignments from 6 different cuts of the dendrogram in Figure 4. The column labels in the file are defined as follows:

`label`	drug-gene pair
`cluster`	cluster assignment for cut height 0.55
`id`	tip ID for the drug-gene pair (no relationship to IDs in other supplements)
`c0.4`	cluster assignment for cut height 0.4
`c0.3`	cluster assignment for cut height 0.3
`c0.25`	cluster assignment for cut height 0.25
`c0.1`	cluster assignment for cut height 0.1
`c0.05`	cluster assignment for cut height 0.05
`cluster.ordered`	cluster assignment for cut height 0.55, corresponding to label in Figure 5
`count`	number of times drug-gene pair in label are co-mentioned in sentences
`pgkb`	binary: 1 if known PGx association, 0 if not
`drugbank`	binary: 1 if known drug-target association, 0 if not
`drug`	the name of the drug from the drug-gene pair
`gene`	the name of the gene from the drug-gene pair
