# Supplementary material for: Learning the Structure of Biomedical Relationships from Unstructured Text
Source: PLoS Comput Biol. 2015 Jul 28;11(7):e1004216. doi: 10.1371/journal.pcbi.1004216 (PMC4517797; doi:10.1371/journal.pcbi.1004216)
Supplement: S4 Data — (PDF) [file pcbi.1004216.s007.pdf]

Supplement F: Prediction certainties from Figure 5 for PharmGKB and DrugBank

We include two files with EBC's scores for the PGx and drug-target prediction tasks on the dense matrix. These are the raw scores used in the generation of the dendrogram in Figure 5. The names of the files are:

`pgx-predictions.tsv`
(PGx prediction scores)

`drugtarget-predictions.tsv`
(drug-target prediction scores)

The columns within these files are:

`drug-gene-pair score indicator-if-known`

The last column, obviously, is meaningless, since none of the pairs in these files are known PGx or drug-target relationships.
